# Integrated optical isolators for broadband multi-laser operation

**DOI:** 10.1038/s41566-026-01978-0

**Published:** 2026-08-13

**Authors:** Kyunghun Han, Yiliang Bao, Junyeob Song, David A. Long, Sean Bresler, Daron Westly, Jason Gorman, Thomas LeBrun, Kartik Srinivasan, Vladimir Aksyuk

**Affiliations:** 1 Microsystems and Nanotechnology Division, National Institute of Standards and Technology, Gaithersburg, MD USA; 2 Theiss Research, La Jolla, CA USA; 3 Whiting School of Engineering, Johns Hopkins University, Baltimore, MD USA; 4 University of Maryland, College Park, MD USA

**Keywords:** Optoelectronic devices and components, Microwave photonics, Silicon photonics, Photonic devices

## Abstract

Photonic integrated circuits commonly feature visible or near-infrared lasers that are vulnerable to destabilizing back-reflections and must be protected by isolators—non-reciprocal optical components enforcing one-way light propagation. Despite recent progress, high-performance isolators remain bulky off-chip components, while on-chip implementations suffer from challenging fabrication, high optical absorption or narrow optical bandwidth. Here we propose and experimentally demonstrate a magnet-free, intrinsically broadband travelling-wave isolator built from foundry-compatible components. Using radio-frequency electro-optic modulation to create synthetic motion across four parallel waveguides, we realize dynamic rotating destructive interference that continuously cancels backward-propagating light while leaving forward-propagating light unaffected. We reach ~30 dB peak isolation, maintain >24 dB isolation across a 30-nm-wavelength span with thermo-optic adjustment and show >20 dB isolation for two lasers simultaneously within 10 nm without any adjustment. The demonstration’s 770–800-nm-wavelength span covers key alkali atomic transitions, enabling on-chip laser isolation for atomic spectroscopy, laser cooling and locking applications. Our isolator approach, applicable from the visible to telecom wavelength spectrum, offers a compelling practical solution, opening the way for fully integrated atomic clocks, quantum sensors, advanced telecommunications and tunable laser systems on a single chip.

## Abstract (web summary)

Radio-frequency electro-optic modulation enables a broadband integrated optical isolator that is magnet-free.

## Main

Photonic integrated circuits (PICs) bring complex optical functionalities on a single chip, enabling applications from precision spectroscopy^1,2^ to quantum sensing^3^ and high-bandwidth communication^4^. Recently, ever more stable, low-noise and tunable laser sources have been integrated on PICs^5,6^. This development highlights the need for integrated optical isolators for protecting such laser sources and ensuring signal integrity. Even tiny back-reflections can destabilize such lasers, causing noise, frequency drift and mode hops, corrupting sensing and communication signals. Free-space magneto-optical Faraday isolators solve this on an optical bench, but they use magnets and magneto-optic materials that are currently impractical for wafer-scale integration. So far, there has been no PIC- and foundry-friendly, magnet-free isolator that is both optically broadband and low-loss for forward transmission.

Unlike electronic diodes leveraging the p–n junction nonlinearity, an optical isolator must use some mechanism to break the Lorentz reciprocity inherent in most optical media^7^. Generally, Lorentz reciprocity can be broken in nonlinear optical systems, but dynamic reciprocity hinders true isolation^8^. In linear optical systems, reciprocity for light can be broken by coupling it to external non-time-reversal-symmetric degrees of freedom through magneto-optic, electro-optic, acousto-optic and thermo-optic effects. An ideal PIC isolator must strongly block the backward-travelling light, add no attenuation to forward-propagating light, operate in the linear single-mode regime independent of optical power level, and provide strong isolation over a wide spectral range. Traditional magneto-optical isolators^9–15^ satisfy these requirements by using the Faraday effect materials in a static magnetic field, resulting in a non-symmetric optical permittivity tensor^15^. However, on-chip integration is hindered by incompatibility with complementary metal–oxide–semiconductor and integrated photonics microfabrication, complexity of large-scale heterogeneous integration and limited optical performance of thin-film magneto-optical materials, particularly increased absorption in the visible range^16^. This motivates the search for practical magnet-free PIC-compatible architectures leveraging acoustic or radio-frequency (RF) travelling waves (TWs).

TW isolators based on plasma dispersion effect^17–23^, electro-optic effect^24–26^ and acousto-optic effect^27–32^ impose spatiotemporal refractive index changes along the waveguides (Extended Data Table 1). In the simplest case of a single-mode waveguide under phase modulation, a driving RF wave travels opposite to the forward-propagating light, leaving the forward light unaffected. Meanwhile, the modulation depth of the co-propagating backward light is adjusted to fully suppress the optical carrier tone, transferring its energy into the harmonic sidebands within the same waveguide. Carefully tuned narrow-band optical filters must be used to remove the sidebands, adding insertion loss and system complexity. Unlike their magnetic counterparts, such single-waveguide TW isolators^33^ have a severely restricted isolation bandwidth, making them impractical for broadband applications involving laser wavelength tuning or fast direct modulation, such as for spectroscopy and multi-channel optical communication.

Another type of TW isolator exchanges optical power between two spatial modes within the same waveguide through modulation-induced mode coupling^17–19,27–31^. This approach operates at special optical frequencies where the two waveguide modes and the TW are frequency- and phase-matched, restricting the isolation centre frequency and limiting bandwidth. Previous work^20,21,23,25^ has explored using multiple waveguide modes in parallel single-mode waveguides, each modulated individually, to enhance performance. However, until now, it has not been known whether it is possible in principle to achieve complete and broadband TW isolation while leaving the forward-propagating light unaffected. All previous schemes exhibited fundamentally unavoidable drawbacks, such as a substantial optical power remaining in unwanted modulation harmonics^23^, degraded isolation performance due to the fundamentally finite rise and fall times of the RF waveform^25^, or a substantial transmission loss for the forward-propagating light due to unavoidable modulation^20^.

The heterogeneous integration method we employ for electro-optic modulation is intrinsically suited for mass-production foundries. Alternatives relying on full thin-film lithium niobate (TFLN) wafers as the starting substrate present practical limitations for large-scale production, primarily due to high material costs and the strict constraints on wafer selection limiting co-integration with electronic integrated circuits. Our PIC platform circumvents these bottlenecks by introducing TFLN strictly during the back-end-of-line compatible bonding phase. In our approach, the underlying Si_3_N_4_ (SiN) PIC can be manufactured in high volume using well-established commercial foundry process flows. From a foundry perspective, handling TFLN solely at the back-end-of-line bonding stage introduces minimal disruption to existing, mature fabrication process flows, providing a practical and flexible route towards mass production.

**Extended Data Fig. 1:**
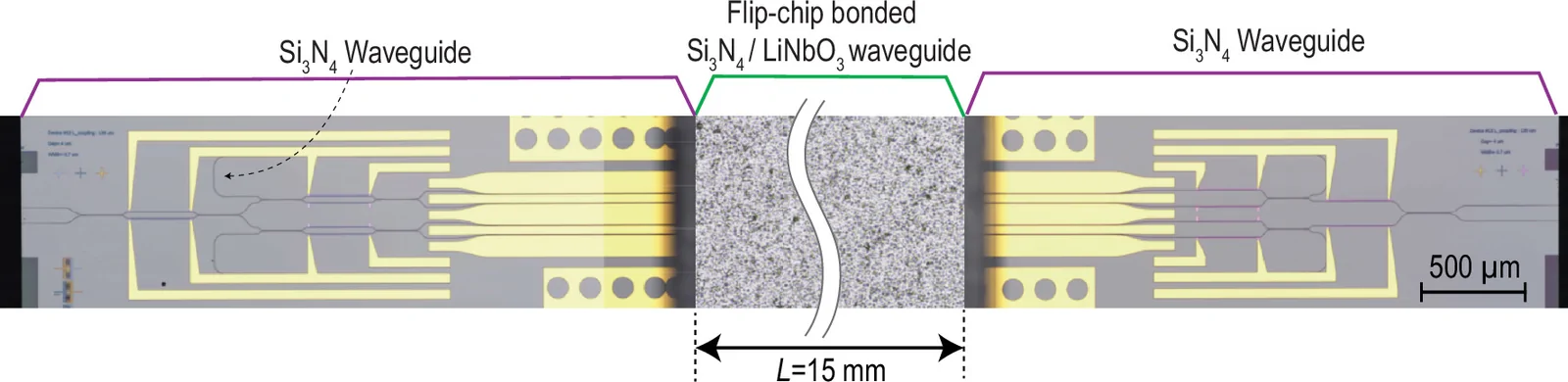
Stitched optical microscopy image of the fabricated isolator. Black narrow horizontally spanning lines indicate silicon nitride optical waveguides. Adjacent chromium microheaters tune the power balance and phase differences among the four parallel channels in the chip’s central region, where flip-chip bonded lithium niobate couples to the silicon nitride waveguide to form an electro-optically active hybrid waveguide. The coplanar RF waveguide carrying the RF driving waves modulates each channel’s optical phase over a length of approximately 15 mm.

Here, we propose and experimentally demonstrate an integrated TW broadband optical isolator achieving a forward-to-backward peak isolation of ~30 dB at a fixed wavelength of 789.7 nm, broadband isolation exceeding 24 dB across a spectral bandwidth of 30 nm (770–800 nm; 14.6 THz) with thermo-optic optimization, and simultaneous isolation for two optical sources within a 10-nm-wavelength window (4.8 THz) exceeding 20 dB without any thermo-optic adjustment. The TW solution to integrated broadband isolation is implemented in the wavelength range particularly important for suppressing feedback-induced laser frequency noise and instability during spectroscopy or locking to alkali atomic transitions such as ^85,87^Rb and ^39^K. Using a four-channel linear RF TW electro-optic phase modulator, our approach establishes the dynamic rotating destructive interference (DRDI), achieving continuous destructive interference under the time-periodic phase modulation. The DRDI scheme fully and continuously suppresses the backward optical power and therefore generates no detectable sidebands in the optical spectrum. The isolator architecture is material-agnostic and is composed entirely of conventional PIC components. We implemented it in a well-established, optically broadband and foundry-compatible SiN and LiNbO_3_ electro-optic PIC material system (Extended Data Fig. 1). Because the isolator does not rely on any resonant structures or mode dispersion engineering, its design is wavelength-scalable from the visible to the telecommunication band, limited only by the bandwidth of passive optical components such as splitters and combiners. Our work experimentally demonstrates compact, efficient, and broadband non-reciprocity through electro-optically induced synthetic motion^34,35^ and provides a practical solution to protect on-chip lasers and other reflection-sensitive photonic components in a fully integrated platform.

## Results

**Fig. 1:**
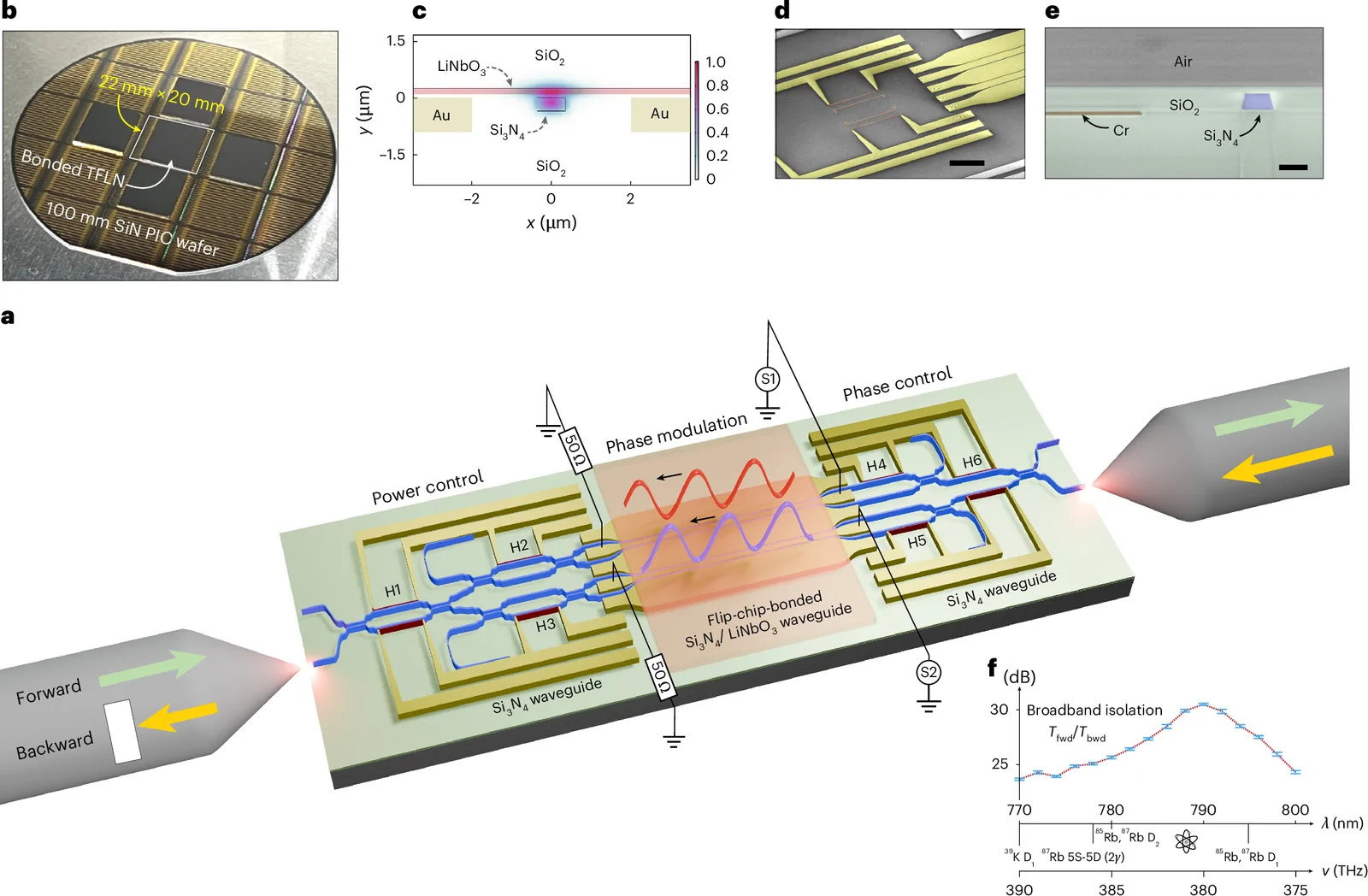
Integrated broadband TW optical isolator. **a**, Graphical illustration of the isolator, showing three functional regions: the left side for power control (H1–H3) for balancing channel optical powers, the centre for TW electro-optic phase modulation (red-shaded region depicting the TFLN layer) and the right side for phase control (H4–H6). Blue-coloured waveguides form the optical path. Gold-coloured metal traces form the electrical paths for driving d.c. currents of integrated heaters (H1–H6) and RF waves (S1 and S2) of the coplanar RF waveguides (terminated by 50 Ω loads). **b**, Photograph of a 100-mm-diameter SiN PIC wafer after bonding TFLN pieces. The white-outlined chip, measuring 22 mm × 20 mm and containing 19 optical isolators, can be fabricated as nine separate dies on a single 100-mm-diameter wafer. **c**, Simulated optical waveguide mode profile showing that the TE mode is confined within the SiN waveguide and TFLN layer surrounded by the silicon dioxide cladding, providing strong overlap in lithium niobate for efficient electro-optic modulation while keeping metal layers outside the optical mode to avoid absorption loss. **d**, Bird’s-eye view false-coloured scanning electron microscopy (SEM) image of the region near H2 and H3 showing the gold RF and d.c. electrical lines (yellow) with Cr microheaters (red). Scale bar, 200 μm. **e**, False-coloured SEM cross-section image of the SiN-only waveguide section showing the SiN waveguide surrounded by SiO_2_ with the Cr microheater (red) horizontally offset by 1.75 μm. Scale bar, 500 nm. **f**, Experimental optical isolation measured over the wavelength from 770 nm to 800 nm. The plotted values represent the mean isolation ± one standard deviation statistical uncertainty of the mean from five wavelength-sweep measurements on the same isolator device, with one isolation value extracted at each wavelength from each sweep. This broadband isolator enables laser stability on multiple alkali atomic transitions such as those used in atomic clocks and laser cooling by suppressing unwanted back-reflections across this spectrum.

**Extended Data Fig. 2:**
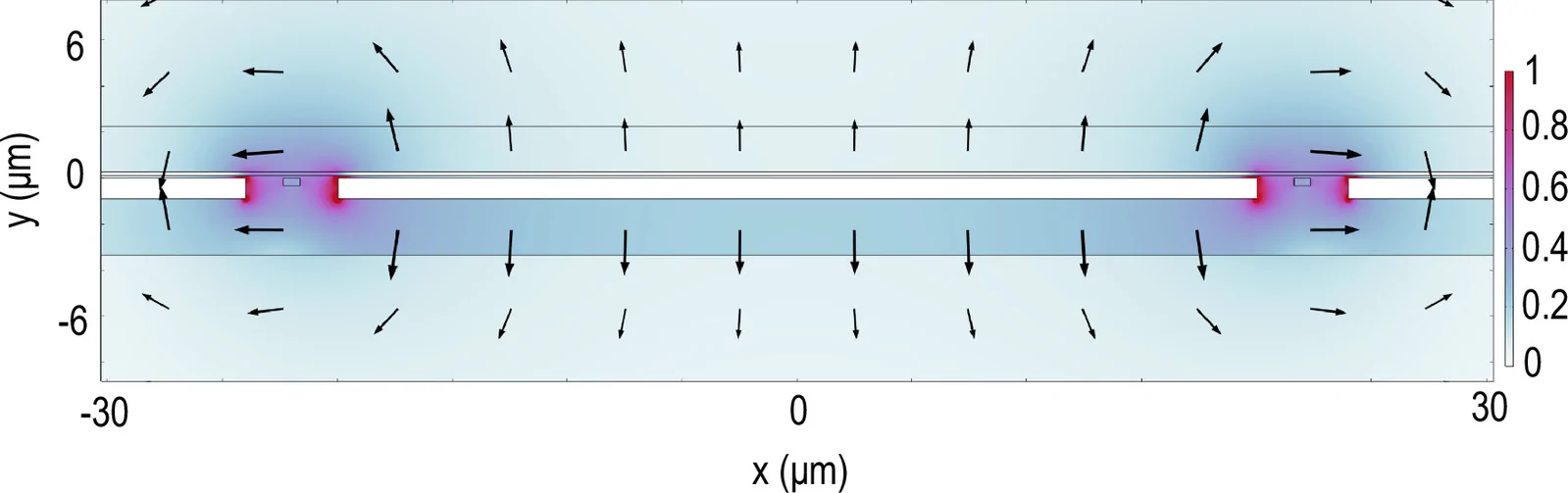
Electrostatic field at the center electro-optic modulation area. Simulated electric field amplitude and vector distribution (black arrows) for a push-pull coplanar RF waveguide. The center buried electrode serves as a signal line, with adjacent ground lines. The electric field is concentrated at the electrode gap edges. The arrow directions indicate opposite electric field polarities between the push-pull pair channels.

We implement the broadband isolator on a ~350-nm-thick silicon nitride (SiN) PIC. The isolator uses a four-channel Mach–Zehnder modulator (MZM), providing four parallel interferometric paths (Fig. 1a). The light with transverse-electric (TE) polarization enters the input waveguide and is split into these four channels by cascaded directional couplers. An X-cut TFLN layer with a thickness of ~150 nm is directly flip-chip bonded onto the passive SiN waveguides to introduce electro-optic modulation, as indicated by the red-shaded region in Fig. 1a. SiN/TFLN hybrid waveguide modes are phase-modulated by RF waves. These waves are applied via a gold coplanar RF waveguide (CPW) with a thickness of 900 nm buried beneath the TFLN (Extended Data Fig. 2). Two separate RF signals are applied to the two push–pull modulator pairs with the CPW lateral electrode gaps of ~4 μm centred on the hybrid waveguides. Figure 1b shows a photograph of a 100-mm-diameter SiN PIC wafer after TFLN bonding, demonstrating that nine 22 mm × 20 mm chips, containing 171 isolators in total, can be fabricated on a single wafer. The simulated optical mode profile of the SiN/TFLN hybrid waveguide confirms that the fundamental TE mode remains confined in the electro-optically active region, providing strong overlap with the TFLN while keeping the metal electrodes outside the optical mode to avoid absorption loss (Fig. 1c). Elsewhere, SiN-only waveguides surrounded by SiO_2_ guide the light, and thermo-optic phase shifters are implemented using embedded Cr resistive microheaters (Fig. 1d,e). The six quasi-static phase shifters (H1–H6) are arranged for accurate tuning of each channel’s optical power and phase. H1–H3 adjust the power balance through the cascaded directional coupler pairs^36^, and H4–H6 adjust the relative phase between the four modulated channels.

When all four channels are set at equal amplitude and in-phase at the output waveguide, forward transmission is maximized, and backward transmission at the input waveguide is suppressed under the appropriate RF waveform. The resulting experimental optical isolation is shown in Fig. 1f, giving ≥24 dB isolation over a ~30-nm-wavelength bandwidth. The isolation is defined in this Article as a ratio of time-averaged optical transmission in the forward direction (*T*_fwd_) to that in the backward direction (*T*_bwd_). Although the isolation principle applies generally for any wavelength, we chose the 770–800 nm range to support future integration for on-chip atomic spectroscopy^37^, quantum sensing^38^ and laser stabilization^39^, operating at key atomic transitions such as ^85,87^Rb D_2_, D_1_ and two-photon lines. Suppression of back-reflections is critical for precise wavelength tuning and stable frequency locking, and it is essential for integrated atomic clock and laser cooling systems that require precise stabilization of multiple lasers.

**Fig. 2:**
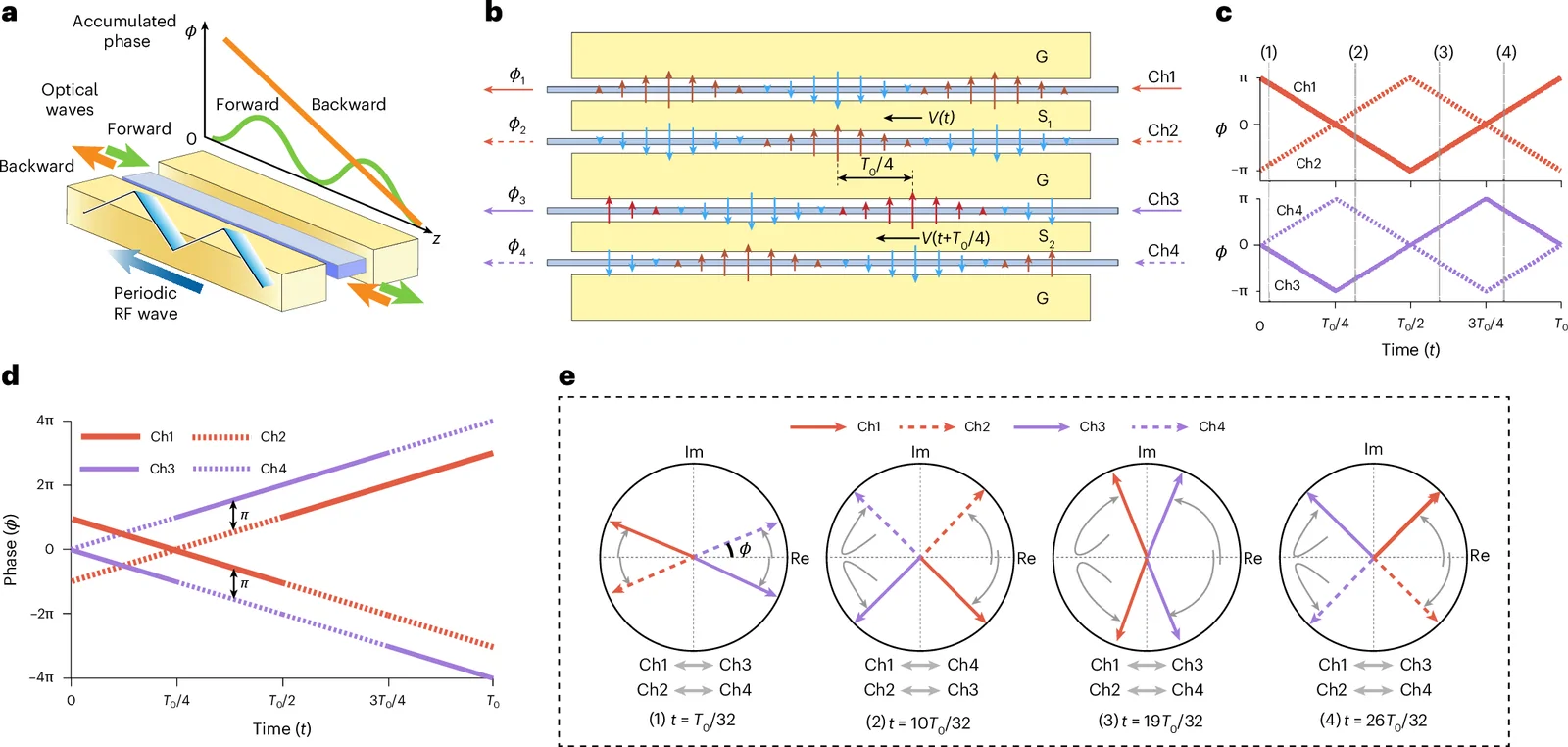
DRDI with four-channel phase modulation. **a**, Conceptual illustration of the single TW phase-modulator element. A periodic RF wave (blue arrow) co-propagates with a backward-propagating light (orange arrow) but counter-propagates relative to a forward-propagating light (green arrow). The plot on the side shows the accumulated phase (*ϕ*) as a function of propagation distance for each direction, illustrating zero net phase shift for the forward-propagating light and linear phase accumulation for the backward-propagating light. **b**, Top-view schematic of the four waveguide channels (Ch1–4) with coplanar RF waveguides (G–S_1_–G–S_2_–G). Push–pull modulation is applied to Ch1 and Ch2 by S_1_ = *V*(*t*) and to Ch3 and Ch4 by S_2_ = *V*(*t* + *T*_0_/4), a quarter-period-delayed waveform. Coloured arrows between electrodes represent instantaneous electric-field vectors. **c**, Accumulated phases of the four channels over one RF period (*T*_0_) with a peak phase modulation of π. **d**, Time-segmented, unwrapped phases from **c**. At any given time, two pairs of channels each maintain a π phase difference and cancel each other. **e**, Phasor diagrams at time instances (*t* = *T*_0_/32, 10*T*_0_/32, 19*T*_0_/32, 26*T*_0_/32), corresponding to vertical dashed lines in **c**, illustrating the rotating cancellation basis. Two channel vectors (one from the upper push–pull pair coloured red and one from the lower push–pull pair coloured purple) are opposite, cancelling each other. These cancellation pairs rotate each quarter period, ensuring seamless destructive interference of the backward-propagating light under dynamic phase modulation.

For TW optical isolators, non-reciprocity arises from breaking the time-reversal symmetry of the optical medium through spatiotemporal modulation of the refractive index along the waveguides, induced by RF waves. When the RF wave propagates along an electro-optic phase modulator (Fig. 2a), optical waves travelling in co-propagating and counter-propagating directions experience different accumulated phases, as described by

1 $${\phi }_{{\rm{FWD}}}(t)=\gamma \int_{0}^{L}{e}^{-\frac{\alpha (L-z)}{2}}{V}_{{\rm{RF}}}\left(t+\frac{z}{{v}_{{\rm{opt}}}}-\frac{L-z}{{v}_{{\rm{RF}}}}\right)\,{\rm{d}}z$$

2 $${\phi }_{\mathrm{BWD}}(t)=\gamma {\int }_{0}^{L}{{\rm{e}}}^{-\frac{\alpha (L-z)}{2}}{V}_{\mathrm{RF}}\left(t+\frac{L-z}{{v}_{\mathrm{opt}}}-\frac{L-z}{{v}_{\mathrm{RF}}}\right)\,{\rm{d}}z,$$

where *z* indicates the position along the modulator, *L* indicates the modulation length and *ϕ*_FWD_ denotes the accumulated phase of the forward-propagating light, which counter-propagates relative to the RF wave. In this case, the optical wave travels from *z* = 0 to *z* = *L*, and the RF wave travels from *z* = *L* to *z* = 0. *ϕ*_BWD_ represents the accumulated phase of the backward-propagating light, which co-propagates with the RF wave from *z* = *L* to *z* = 0. *V*_RF_(*t*) refers to the instantaneous RF voltage waveform at a time *t*. *α* is the power attenuation constant of the RF wave, *v*_RF_ is the phase velocity of the RF wave and *v*_opt_ is the group velocity of the light. *γ* is the electro-optic phase modulation coefficient.

When the forward-propagating light interacts with a counter-propagating RF wave with a zero-mean periodic waveform, the instantaneous phase modulation experienced by the light is itself a zero-mean periodic function. In the limit of negligible RF power attenuation (*α* ≈ 0), choosing

3 $${f}_{\mathrm{RF}}=\frac{m}{\frac{L}{{v}_{\mathrm{RF}}}+\frac{L}{{v}_{\mathrm{opt}}}},$$

where *m* is a positive integer, makes the total interaction time ${t}_{{\rm{int}}}=\frac{L}{{v}_{{\rm{RF}}}}+\frac{L}{{v}_{{\rm{opt}}}}$ equal to integer multiples of RF periods, resulting in zero net phase accumulation over the modulation length *L*. Under this condition, the forward-propagating light passes through the modulation region without experiencing any net phase shift due to the RF signal.

While achieving this condition only requires adjusting the RF frequency, suppressing the backward-propagating light is far more challenging. This difficulty arises from the nature of phase modulation, which inherently generates an infinite series of frequency harmonics. Achieving complete destructive interference for the backward light requires careful consideration of the phase relationships among all harmonic components with different phases. Identifying an optimal RF waveform consisting of multiple frequency components that leads to complete destructive interference is analytically non-trivial. For example, phase modulation is described by Bessel functions of the first kind, which, unlike polynomials, do not lead to simple closed-form solutions for harmonic cancellation. Therefore, rather than seeking a solution purely from a frequency domain perspective, it is more effective to consider the time domain perspective.

**Extended Data Fig. 3:**
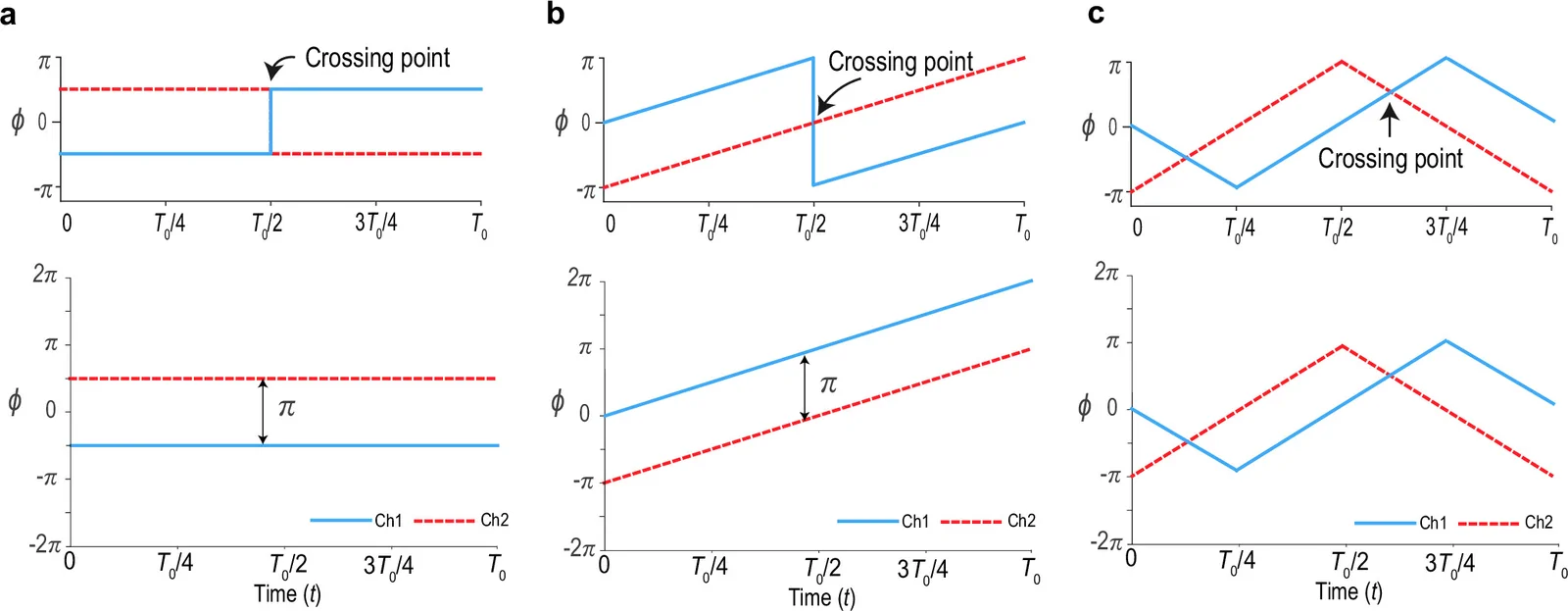
Accumulated phase for two time-delayed RF drive waveforms. **a**-**c**. Accumulated optical phase (top row) and the same phase traces segmented and aligned in time (bottom row) over one RF period for three waveform shapes are presented. **a**. Square waveform gives an ideal constant *π* phase difference over each half period, resulting in continuous destructive interference but requires infinite transition slopes at every half periods. Finite transition slopes introduce partial destructive interference and reduce isolation performance at the phase crossing points. **b**. Sawtooth wave produces linear phase ramps but requires infinite transition slopes as well, leading to the same limitation in isolation performance. **c**. Triangular waveform has both positive and negative slopes which can eliminate the infinite transition slope but produce the unavoidable phase crossing points where two phases become equal, preventing continuous destructive interference throughout the RF period. The DRDI can overcome this limitation by introducing two additional RF waves and periodically rotating the canceling pairs so that each pair maintains a continuous destructive interference.

The fundamental principle for blocking coherent waves is destructive interference, which occurs when two or more waves combine so that their phasor vectors sum to zero. In practice, this can be achieved by combining waves of equal amplitude with constant phase difference over time. However, maintaining a constant phase difference between the coherent waves becomes challenging under periodic zero-mean phase modulation, which is required to keep the forward-propagating light unaffected. One simple possibility is to drive the phase modulation with two square waves or sawtooth waves with a half-period delay to mimic the constant-phase condition using only two optical waves^25^. However, the abrupt waveform discontinuities inherent to these waveforms make this method impractical and substantially limit the achievable isolation performance (Extended Data Fig. 3). By contrast, triangular waveforms do not have waveform discontinuities in the time domain because they exhibit both positive and negative slopes, although discontinuities exist in their derivatives. Because triangular waveforms include both slope polarities, destructive interference between two phase-modulated waves is impossible. There is always a crossing point at which their modulated phases coincide. We therefore developed a DRDI scheme to avoid this issue and achieve complete cancellation of phase-modulated waves using four triangular or, more generally, four time-delayed waveforms with both positive and negative slopes. Later in this Article, we introduce a waveform design procedure to remove derivative discontinuities inherent to triangular waveforms, while optimizing for higher isolation. Here, we use the triangular wave to describe the underlying principle.

To implement the DRDI with the triangular wave, we use a four-channel electro-optic phase modulator with a G–S_1_–G–S_2_–G coplanar RF waveguide driven by two RF waves (Fig. 2b). Channel (Ch)1 (Ch1) and Ch2 form an upper push–pull pair, while Ch3 and Ch4 form a lower push–pull pair. Within a pair, two channels are driven by the same RF wave but opposite polarity (Extended Data Fig. 2). Furthermore, driving RF waves for the two push–pull pairs have time delay by one-quarter period (*T*_0_/4) relative to each other. This configuration simplifies an experimental set-up for equally spaced time delays of 0 (Ch1), *T*_0_/4 (Ch3), *T*_0_/2 (Ch2) and 3*T*_0_/4 (Ch4). When the phase modulation is driven by a triangular waveform whose peak-to-zero voltage amplitude equals the half-wave voltage, *V*_π_, the accumulated phases (*ϕ*) across the four optical channels are shown in Fig. 2c. To understand this mechanism more intuitively, we can time-segment and unwrap the accumulated phases (*ϕ*_1_, *ϕ*_2_, *ϕ*_3_, *ϕ*_4_) over time (Fig. 2d). This reveals a structure in which, at any given moment, there are always two wave pairs that maintain a relative phase difference of π, ensuring continuous destructive interference. In phasor space, this corresponds to a dynamic rotation of cancellation pairs at every quarter period, sustaining destructive interference throughout the RF wave period (Fig. 2e). Ch1 is cancelled by Ch3 during 0 ≤ *t* < *T*_0_/4, by Ch4 during *T*_0_/4 ≤ *t* < *T*_0_/2, and so on. Similarly, Ch2 alternates its cancellation pair between Ch4 and Ch3 through the RF cycle. By rotating the destructive interference pairs in time, the overall waveform maintains a zero-mean condition, with both positive and negative slopes in the phase modulation, eliminating abrupt discontinuities found in the square and sawtooth waveforms. To visualize this DRDI more intuitively, an animated version of the phase dynamics and rotating cancellation basis is provided in Supplementary Video 1.

The triangular waveform represents a particular solution within a broader class of waveforms that satisfy the conditions for the DRDI. The general requirements for the driving RF waveform are further discussed in the Supplementary Information. In principle, DRDI can achieve arbitrarily high isolation under perfect amplitude and phase balances with sufficient harmonics. This represents a key advantage over resonance-based isolators which fundamentally must trade operational bandwidth for isolation. The DRDI imposes no such inherent compromises beyond the technological constraints of waveform generation and device uniformity.

**Extended Data Fig. 4:**
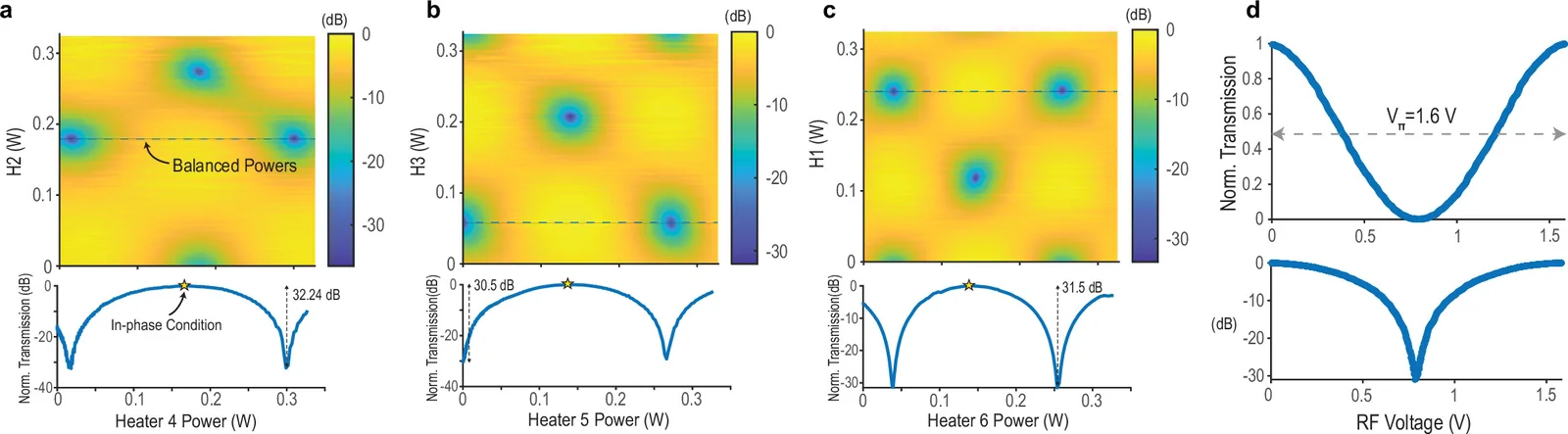
Characterization of integrated heaters and electro-optic half-wave voltage. **a**-**c**. (Upper) 2D maps of normalized optical transmission with no RF in logarithmic scale as a function of heater electrical power for the upper channel pair (H2, H4), the lower channel pair (H3, H5), and the inter-pair (H1, H6), respectively. H1, H2, and H3 control power balance between the channels while H4, H5, and H6 control phase relationship between the channels. Dashed lines indicate the heater settings balancing optical powers between the optical channels. (Lower) Normalized optical transmission plotted in logarithmic scale as a function of the heater electrical power at the dashed lines, showing static extinction ratios of 32.24 dB (Ch1 and 2), 30.5 dB (Ch 3 and 4), and 31.5 dB (Ch1-4). The laser wavelength was set to approximately 790 nm. **d**. (Upper) Normalized optical transmission through a single push-pull pair as a function of low-frequency RF drive voltage, indicating a single-arm modulation half-wave voltage at ≈ 1.6 V. (Lower) Same data plotted on a logarithmic scale.

The integrated microheaters are not strictly necessary for the proposed optical isolator, as the intrinsic phase shifts of the broadband 1 × 4 optical splitters and combiners can be perfectly compensated by incorporating passive optical delay lines. However, to compensate for experimental variations, we utilize thermo-optic effects in the SiN core and SiO_2_ cladding to balance channel powers and set static phase offsets (Extended Data Fig. 4). The left-side heaters (heaters 1–3) balance the optical powers of all four optical channels, compensating for any residual splitting asymmetry introduced by the directional couplers as well as propagation loss differences. Heaters 2 and 4 tune the relative optical powers and optical phases of Ch1 and Ch2. Heaters 3 and 5 do the same for Ch3 and Ch4. Heaters 1 and 6 then adjust the relative optical powers and phases between the combined optical waves from the upper (Ch1 and Ch2) and lower (Ch3 and Ch4) push–pull pairs. By fixing heater 1 such that only the upper pairs carry optical power, and sweeping heaters 2 and 4, we map the thermo-optic tuning response of that pair. The wavelength of the laser is centred at approximately 790 nm. At a d.c. power change of 0.28 W, we achieve a full 2π phase shift, showing two transmission minima when the optical powers of the channels are balanced. Even when the heaters are driven with 0.35 W of electrical power, no noticeable thermal drift is observed during measurement. Measured static extinction ratios are ≥32 dB (Ch1 and Ch2), ≥30 dB (Ch3 and Ch4) and ≥31 dB for all four channels combined.

These static extinction ratio measurements define the upper experimental limit of isolation performance for the electro-optic case, as both thermo-optic and electro-optic interference rely on the same linear optical behaviour of the chip. In practice, electro-optic isolation may be further degraded by RF attenuation, velocity mismatch, finite electrode bandwidth, impedance mismatches and parasitic reflections, so it cannot exceed the static thermo-optic bound. With optimized coupler designs, such as multimode-interference couplers^40,41^ and subwavelength-assisted directional couplers^42^, the static extinction ratio and bandwidth can be further improved. For the DRDI operation, all four channels must be combined in-phase at the output waveguide in the absence of the RF drive. Our protocol for finding the balanced powers and in-phase setting is described in the Supplementary Information. We characterize the electro-optic modulators by applying a low-frequency RF to the CPW while injecting light into one push–pull pair. A half-wave voltage (*V*_π_) of a single-arm phase modulator is measured at ~1.6 V, which is twice that of the push–pull case. The extinction ratio under low-frequency RF closely matches the thermo-optic value, confirming that the thermo-optic case indeed represents the upper bound for electro-optic isolation performance.

While the physical path lengths of the four parallel optical waveguides are designed to be strictly identical, typical fabrication non-idealities such as lithography dose and waveguide layers’ thickness variations can introduce slight differences in the effective refractive index of the waveguide modes among the optical channels. These discrepancies can cause the in-phase condition to drift with temperature owing to different thermo-optic phase shifts along the modulation region. However, transferring this design to commercial foundry processes with highly calibrated lithography and uniform film deposition will notably decrease these variations. Furthermore, unlike isolators that rely on narrow-linewidth resonant components such as microrings, our Mach–Zehnder interferometer-based design remains intrinsically more robust against temperature-induced performance degradation.

**Fig. 3:**
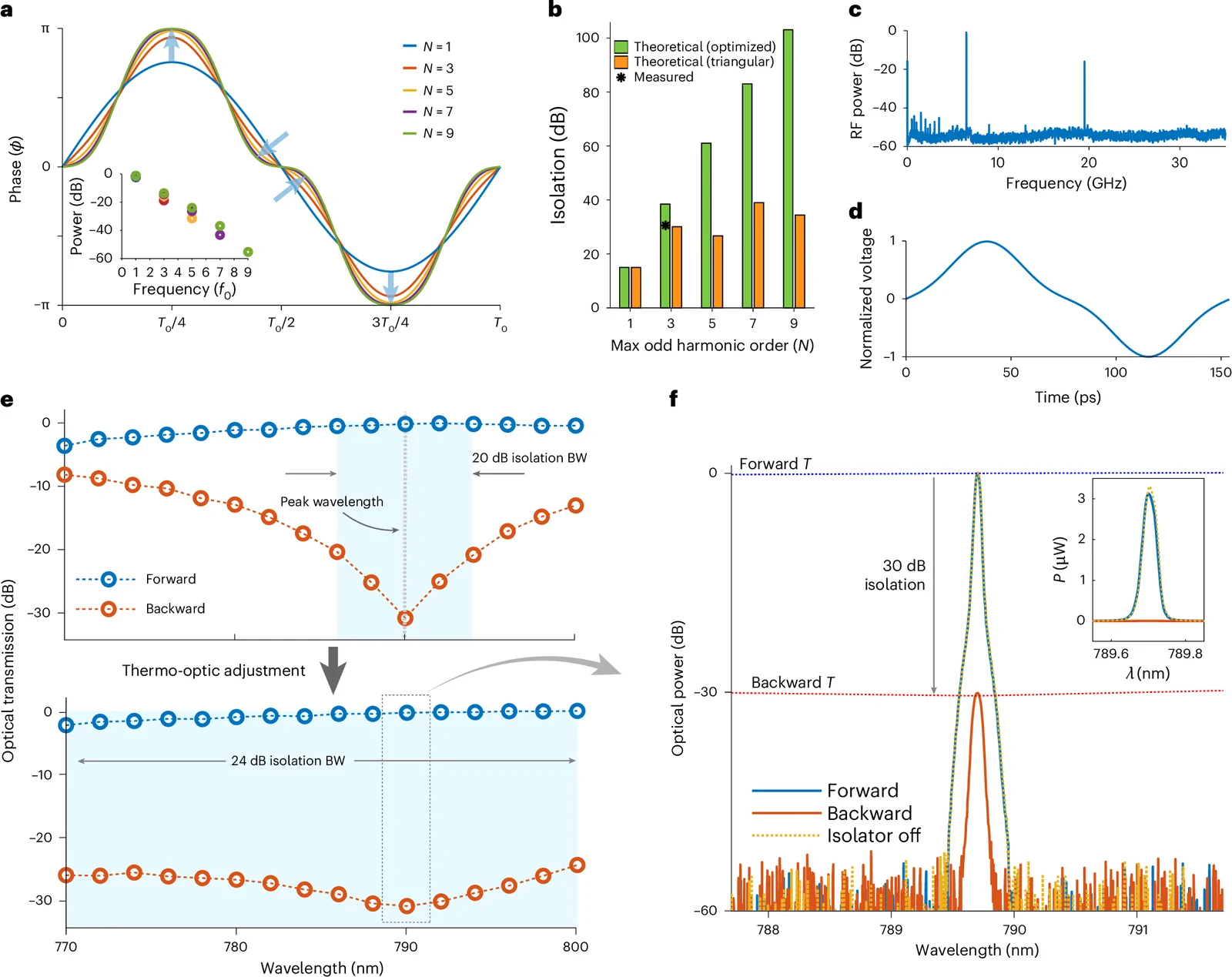
Broadband optical isolation demonstration with an optimized multi-harmonic RF waveform. **a**, Accumulated phase with optimized time-domain waveforms for a finite set of odd harmonics that satisfy half-wave symmetry. The inset shows the corresponding odd-harmonic power coefficients in a logarithmic scale. As the maximum odd-harmonic order (*N*) increases, the peak-to-zero phase amplitude approaches π, and slopes at *t* = 0, *T*_0_/4, *T*_0_/2 and 3*T*_0_/4 approach zero to avoid modulated phase derivative difference to the quarter-period-delayed waveform. **b**, Theoretical isolation versus maximum odd-harmonic order for truncated harmonic triangular waveform (orange) and numerically optimized waveform (green). The experimentally measured isolation is shown as a black star. **c**, Measured RF power spectrum at the CPW output for the predistorted 6.5 GHz optimized waveform, with substantial power only at the fundamental frequency and the third harmonic. Power scale is normalized to the fundamental frequency power. **d**, Normalized voltage time-domain waveform reconstructed by inverse Fourier transform of the spectrum in **c**, showing rounded waveforms at maxima, minima and zero-crossing points. The voltage scale is normalized to its maximum voltage. **e**, Wavelength dependency of normalized optical transmissions (normalized by active forward transmission at a wavelength of 800 nm) for the forward- (blue) and backward-propagating light (red) with a fixed heater setting optimized at a wavelength of 789.7 nm (top) and an actively adjusted heater setting for each wavelength (bottom). The blue-shaded region indicates the isolation bandwidth (BW), which is the wavelength range where the isolations are above 20 dB. With actively adjusted heaters for each wavelength, the isolation enhances >24 dB across the 30-nm-wavelength bandwidth. The measurement uncertainties, primarily caused by fibre-to-chip coupling drift, are estimated to be smaller than the plotted symbol size. **f**, Optical spectra measured near 789.7 nm with the isolator turned on for the forward- (blue) and backward-propagating optical wave (red), showing negligible sidebands. The spectrum of the forward-propagating light without the RF power (yellow) almost matches the forward spectrum with RF power. The optical powers are normalized to the peak power of the ‘isolator off’ spectrum. The time-averaged optical transmissions near this wavelength are plotted as dotted lines for the forward-propagating light (blue) and the backward-propagating light (red). The inset shows the same spectrum with linear-scale optical powers.

**Extended Data Fig. 5:**
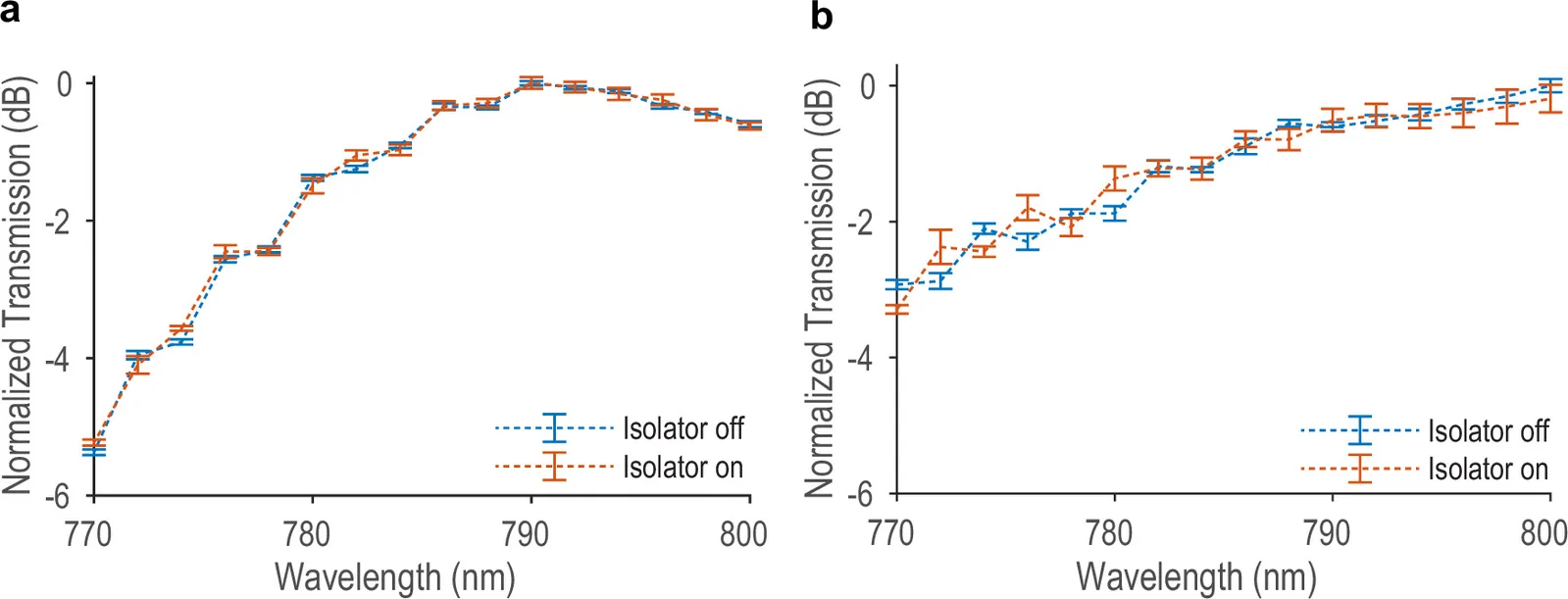
Effect of a 6.5 GHz RF drive on the forward-propagating transmission. Normalized forward transmission is plotted as a function of wavelength from 770 nm to 800 nm. The plotted dashed lines connect the average normalized transmission values obtained from five wavelength-sweep measurements on the same isolator device. At each wavelength, one normalized-transmission value was extracted from each sweep. The error bars indicate ± one-standard-deviation statistical uncertainty of the mean normalized transmission. The plot shows the normalized forward transmission for **a**. a fixed optimization wavelength at 789.7 nm and **b**. actively adjusted and optimized for every wavelength. The blue dotted line and its error bars represent the transmission with no RF, while the orange dotted line and its error bars show the transmission under the 6.5 GHz RF wave. For the fixed optimization case, both lines have a peak transmission at nominal 790 nm which is the optimized wavelength for the microheaters, and the transmission values are normalized to this peak. For the active tuning case, a peak transmission occurs at nominal 800 nm, and the transmission values are normalized to this peak. The two lines overlap at most wavelengths, indicating that the RF wave imposes no additional insertion loss across the wavelength range.

For a practical realization at a base frequency of several gigahertz, the ideal RF waveform must be approximated by truncating its Fourier series to a finite set of harmonics within the available RF waveform generation bandwidth. Without any adjustment, such truncation shifts the system away from the exact DRDI condition and reduces isolation. To maximize isolation for truncated triangular waveforms with odd harmonics up to *N*th harmonics, we use a gradient-descent algorithm to numerically optimize the amplitudes and phases of odd harmonics up to *N*th order, while enforcing half-wave symmetry. For optimizing the waveform, the parameter space is confined to a small number of amplitudes and relative phases. Given the physical constraints of the electro-optic modulation bandwidth and available RF power, we seek a practical multi-tone waveform solution maximizing the isolation within these constraints. Figure 3a plots the accumulated phases with these optimized waveforms for *N* = 1, 3, 5, 7 and 9. As *N* increases, the peak-to-zero phase amplitude (*ϕ*) approaches π, and the slopes at *t* = *T*_0_/4 (peak), *T*_0_/2 (zero-crossing) and 3*T*_0_/4 (dip) become gradually rounded, eliminating the derivative discontinuities of a triangular waveform. The rounded zero-crossing ensures that the derivatives of the modulated phase match that of the quarter-period-delayed waveform, which aligns at its peaks and dips. To maintain perfect cancellation of phase-modulated light at those instants, the phase-modulated waveforms must differ by π and share identical instantaneous slopes to maintain perfect cancellation of the backward-propagating light. The resulting theoretical isolation increases monotonically with *N* (Fig. 3b). With only the first and third harmonics (*N* = 3), the optimized waveform achieves a theoretical isolation of 38 dB. This reduces the complexity and bandwidth requirements of the high-frequency RF system. It is worth noting that this multi-tone modulation strategy relies on the exact integer harmonics. In platforms where generating exact integer harmonics is challenging, the continuous temporal phase walk-off between the non-integer tones can lead to a degradation in isolation. In practice, RF attenuation, velocity mismatch, finite CPW bandwidth and impedance mismatches will further limit the achievable isolation. In the DRDI scheme, applying the correct RF waveform to the isolator is critical because most RF components introduce both linear and nonlinear distortions that scramble harmonic phases and amplitudes. The RF spectrum must contain only odd harmonics to satisfy the half-wave symmetry requirement of the DRDI, *f*(*t* + *T*_0_/2) = −*f*(*t*). We generate a 6.5 GHz RF waveform with an arbitrary waveform generator and then iteratively predistort it by monitoring the RF spectrum at the CPW output to compensate for distortions in the RF system. The frequency of 6.5 GHz is selected for the zero net accumulated phase for the forward-propagating light. This frequency exceeds the theoretical value predicted for the modulation length (*L*) of 15 mm. We attribute this discrepancy to RF attenuation or a lower RF phase index arising from bonding artefacts such as air voids near the CPW. Importantly, the DRDI scheme preserves the transmission of the forward-propagating light without introducing additional measurable loss (Extended Data Fig. 5). To rigorously quantify this, we performed a statistical comparison of the forward transmission spectra in the presence and absence of the RF drive. Across a 30-nm-wavelength span, the application of the RF drive introduces a negligible excess insertion loss of 0.023 dB ± 0.018 dB under a fixed heater bias, and 0.009 dB ± 0.056 dB when the heaters are actively adjusted for each wavelength. These variations are statistically indistinguishable from zero within the tight bounds of small experimental measurement uncertainties.

Due to RF component bandwidth limitations, we truncate the RF waveform at the third harmonic (19.5 GHz). The measured RF spectrum shows no even-order harmonics, and the odd-harmonic powers closely follow the theoretically optimized waveform (Fig. 3c). At the RF CPW output, an RF power meter reads an RF power of 32 mW for one push–pull pair, 64 mW in total. Assuming a matched 50 Ω load, this corresponds to an equivalent sinusoidal *V*_p–p_ ≈ 3.6V. Our low-frequency sinusoidal single-arm *V*_π_ is 1.6 V, so implementing the isolator requires only a swing from −*V*_π_ to +*V*_π_, about 3.2 V_p–p_, which is comparable to the measured voltage. To determine the actual RF drive requirements, the average power measured directly after the RF amplifier was 402 mW per channel. After calibrating for RF cable and probe losses, the on-chip RF insertion loss of the isolator was evaluated to be 8.1 dB. Given this propagation loss and the predistorted multi-tone nature of the driving signal, the required on-chip RF voltage naturally exceeds the low-frequency *V*_π_ estimation due to the frequency-dependent RF *V*_π_ roll-off. In addition to the RF power, the integrated microheaters in our experiment consume between 0.12 W and 0.27 W of d.c. electrical power to balance the optical powers and phase offsets between the channels (Supplementary Fig. 4). These voltages fall within the low few-volt regime that is practical for on-chip PIC. Notably, no optical or RF resonators and no watt-level electronics are required. An inverse Fourier transform of that measured spectrum reconstructs a time-domain waveform that closely approximates the optimized waveform with rounded peaks, dips and zero-crossing points (Fig. 3d). Supplementary Video 2 shows the corresponding accumulated-phase, unwrapped-phase and phasor-space dynamics for the theoretically optimized waveform with *N* = 3 as shown in Fig. 3a.

With this optimized RF waveform, we measured spectra of forward- and backward-propagating light by an optical spectrum analyser (Fig. 3f). The forward-to-backward peak-spectrum power ratio is measured at ~30 dB, which directly approximates the isolation because negligible sidebands above noise exist in the spectra. Moreover, the spectrum of the forward-propagating light under RF is almost identical to the ‘isolator off’ case, which refers to the forward-propagating light with no RF, indicating no additional loss and frequency shift for the transmitted light. The frequency dependence of the forward transmission is discussed in the Supplementary Information. As demonstrated by our RF and optical spectrum measurements for forward transmission, the DRDI scheme is robust against residual phase modulation, with no observable sidebands or other spectral features generated above the measured noise floor. Therefore, substantial degradation of the transmitted light is not expected. However, for applications requiring state-of-the-art frequency stability, such as optical atomic clocks, integrating active feedback loop stabilization may be necessary to further suppress any residual noise.

To characterize wavelength dependence, we sweep the laser wavelength from 770 nm to 800 nm while holding the integrated heaters at their optimal in-phase, balanced power settings (tuned to 789.7 nm) (Fig. 3e). The isolation varies approximately 21 dB across the 30-nm-wavelength span, primarily due to the wavelength-dependent splitting ratio of the parallel waveguide directional couplers. The maximum isolation is measured at 30.6 dB ± 0.1 dB. By adjusting the heater powers separately for each wavelength, we achieve >24 dB isolation over a 30-nm-wavelength span. Replacing these directional couplers with broadband multimode-interference (MMI) or adiabatic designs would further flatten the response and may reduce or eliminate the need for thermo-optic adjustment. To target other wavelength bands such as telecommunication C-band, one could redesign the power splitters and combiners for that wavelength while the DRDI principle itself remains unchanged.

**Fig. 4:**
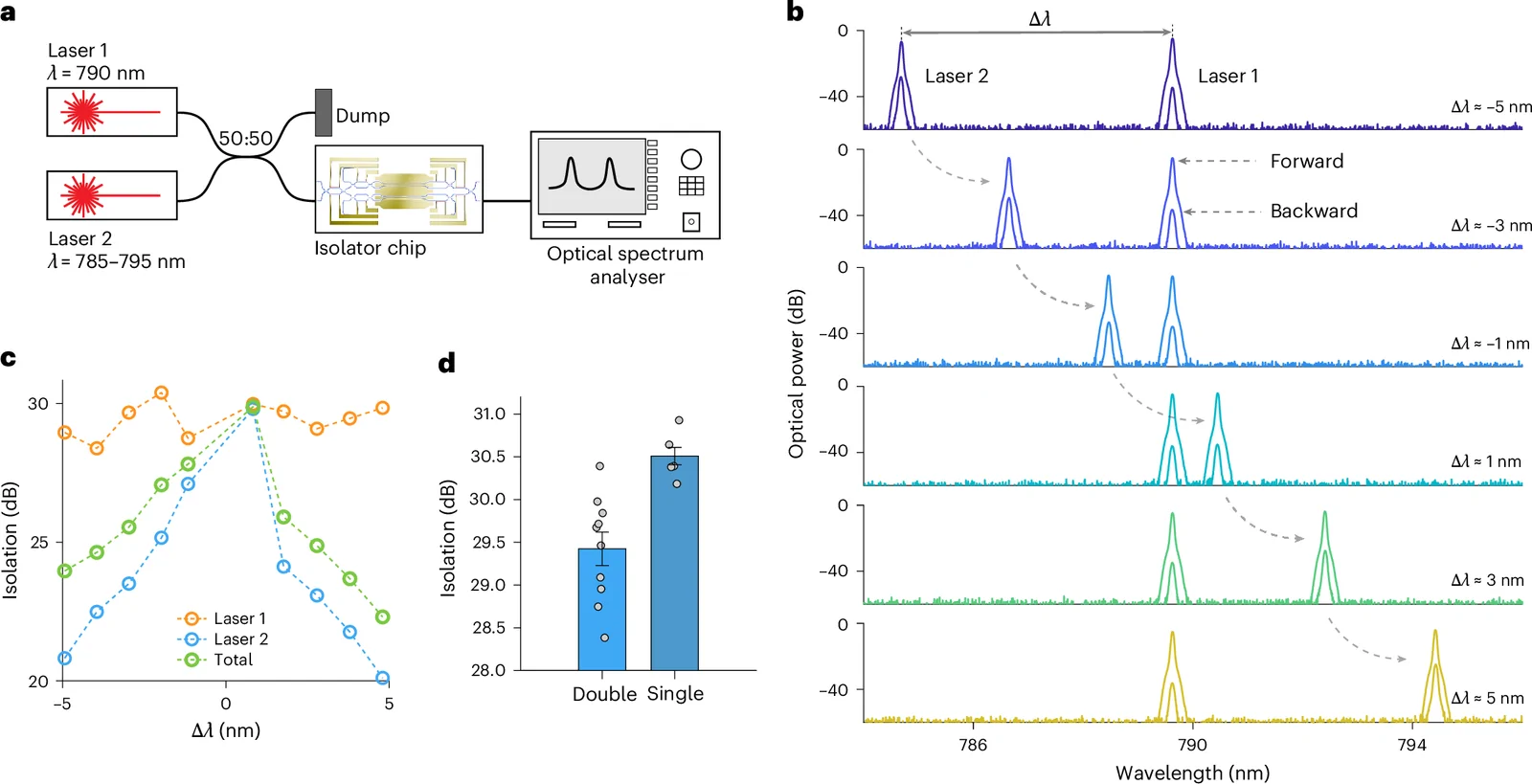
Double-laser experiment for broadband isolation performance. **a**, Experimental set-up showing that two external-cavity diode lasers, laser 1 fixed at *λ*_1_ ≈ 789.7 nm and laser 2 swept through *λ*_2_ ≈ 785 nm to 795 nm, are combined by a 50:50 optical fibre coupler. The combined signal passes through the isolator chip and is analysed on an optical spectrum analyser. For backward transmission, the input and output fibres are physically interchanged. The heaters are optimized for a wavelength near 790 nm. **b**, Forward- and backward-propagating optical spectra for various wavelength difference Δ*λ* = *λ*_2_ − *λ*_1_. In all cases, the backward transmissions for both lasers are suppressed by >20 dB, showing broadband isolation. The optical power scale is referenced to 1 mW. **c**, Extracted measured isolations from the optical spectra as a function of Δ*λ* for laser 1 (orange circles), laser 2 (blue circles) and the combined total signal (green circles). Laser 2 maintains >20 dB isolation across a 10-nm-wavelength sweep, while laser 1 remains near 30 dB with minimal variation. **d**, Comparison of isolations at *λ* ≈ 789.7 nm under single- and double-laser operation. Because of the additional laser, the isolation is reduced by ~1.1 dB and the measurement uncertainty increases. Bars indicate mean isolation values, and error bars indicate ± one standard deviation statistical uncertainties of the mean. Overlaid circles show the underlying data points. Each data point was obtained from an individual wavelength-sweep measurement on the same isolator device, with ten sweeps for the double-laser and five sweeps for the single-laser operation.

To further demonstrate the distinctly broadband nature of our DRDI TW isolator, we rigorously evaluate the isolation performance under simultaneous multi-wavelength operation in a double-laser experiment (Fig. 4a). Two external-cavity diode lasers were combined through a 50:50 fibre coupler, delivering equal optical power from both lasers to the isolator chip. Laser 1 was fixed at a wavelength of 789.7 nm, while laser 2 was swept across the spectral range from nominally 785 nm to 795 nm in 1-nm increments. For each detuning Δ*λ* = *λ*_2_ − *λ*_1_, we measured forward and backward transmission spectra on an optical spectrum analyser by interchanging the input and output fibres. The measured spectra are shown in Fig. 4b. These results show that, in every case, the backward transmission of both lasers is suppressed by more than 20 dB across the entire 10-nm span. From each spectrum, we then numerically integrated the power spectral density over the peak region of each laser in both forward and backward directions to compute the isolation values. The resulting data are plotted in Fig. 4c. As expected, the maximum total isolation occurs near 789.7 nm, corresponding to the heater settings optimized for that wavelength, and decays symmetrically as the wavelength is detuned. This roll-off closely matches the single-laser result discussed in Fig. 3e. Small oscillations in laser 1 isolation indicate minor perturbations introduced by laser 2, despite the theoretical orthogonality of two different wavelengths at low optical power. Figure 4d presents a comparison of mean isolation at 789.7 nm under single-laser and double-laser operation. The double-laser case exhibits 29.4 dB ± 0.2 dB isolation, which is an only approximately 1.1 dB reduction in mean isolation, accompanied by a slight increase in measurement uncertainty. These results confirm that our isolator reliably maintains more than 20 dB isolation over a 10-nm bandwidth for multiple lasers, with negligible crosstalk and without introducing any additional complexity into the RF electronics. These results represent a qualitative advance over earlier TW isolators, which relied on resonant features and were constrained by a narrow isolation bandwidth. The broadband isolation demonstrated here is essential for applications that require stable operation of multiple laser sources such as magneto-optical trapping of neutral atoms and optical frequency comb spectroscopy. Our approach removes resonant linewidth limitations and is not fundamentally cavity-bandwidth-limited. The remaining constraints arise from splitter/combiner dispersion, finite waveform harmonics and RF loss/mismatch.

## Discussion

Unlike any previous magnet-free integrated isolator approaches, the DRDI scheme offers complete broadband isolation without forward loss or modulation, limited only by the physical and technological constraints of the underlying electro-optic PIC platform. In contrast to all previous TW isolators, the DRDI scheme fully and continuously blocks both the carrier tone and all modulation harmonics without exception, and therefore can isolate multiple tones simultaneously, while using a smooth, easily realizable RF waveform.

In addition to its technological importance, the current experimental demonstration advances the rapidly developing field of wave propagation through engineered spatiotemporally varying media^34,35^. The DRDI spatiotemporal modulation of four identical waveguide modes with sequentially and equally time-delayed periodic signals can be interpreted as linear synthetic motion in two dimensions: along the waveguide propagation and ‘laterally’ across the waveguides sequentially indexed according to the time delay. The lateral dimension can be thought of as either translation or rotation: the waveguide index mapping to a linear or an azimuthal coordinate, respectively. The passive coupler sections before and after the synthetically moving media effectively implement a spatial Fourier transform along the lateral dimension. The synthetically moving modulation is transparent for the counter-propagating forward wave, while imparting a lateral wavevector (or an angular momentum), together with a frequency shift, on the backward wave. The specific waveform of the DRDI modulation results in a time-persistent destructive interference null at the output waveguide, which corresponds to the wavevector (angular momentum) of zero. Notably, the shifted light may be recoverable from an alternate waveguide output, whereby the functionality of the device may be extended to that of an optical circulator.

The ~30 dB peak isolation at an optimum wavelength, >24 dB isolation across a ~30-nm-wavelength range with wavelength-dependent thermo-optic adjustment and >20 dB simultaneous isolation of multiple tones across a 10-nm range will immediately enable integrated laser PIC light engine applications in the 770–800 nm range containing commonly used atomic transitions. Having no intrinsic isolation-bandwidth trade-offs, the performance is expected to further improve with RF waveform and directional coupler optimization and more accurate and uniform fabrication. The need for thermo-optic tuning, used here to counteract the fabrication variations and the limited bandwidth of simple passive coupler designs, may be reduced or even eliminated entirely. Applications integrating multiple laser sources may share isolators, or use multiple identical or parametrized isolator designs and share common RF drive power. Future PICs may also leverage the ability to turn the isolators on and off, superimpose forward modulation without affecting backward isolation, and redirect the backward light into an alternate waveguide for processing.

The commonly used, foundry-compatible SiN/LiNbO_3_ fabrication platform is broadly extendable to other wavelengths, from visible to near-infrared. For example, with suitable component geometry modifications, the isolator can be implemented for the telecommunication C-band or other infrared wavelengths. Moreover, the isolator is composed of common electro-optic PIC components, such as waveguides, directional couplers and electro-optic modulators, and therefore is universally compatible with a variety of other platforms, such as the ones using directly patterned thin-film LiNbO_3_, LiTaO_3_ or other electro-optic materials. Requiring only 2π modulation depth, the isolator operates with easily accessible RF voltages and powers, which pose no special drive power requirements. Practically, this isolator can be integrated with tunable lasers or frequency combs, eliminating the need for bulk isolators and enabling more complex and stable on-chip photonic systems. By solving a long-standing bottleneck in PIC design, the lack of a broadband on-chip isolator, our work paves the way for fully integrated photonic devices that can operate with one-way light flow for applications in data networks, quantum technology and precision spectroscopy.

## Methods

### Device fabrication

The device was fabricated on a 100-mm-diameter silicon wafer with a 3-μm wet thermal oxide layer and 350-nm stoichiometric SiN. The SiN waveguides were patterned using an ultraviolet stepper lithography tool and a reactive-ion etcher. First, the microheaters were patterned by the stepper, and then 100 nm of the chromium layer was deposited by electron-beam evaporator tool, followed by a metal lift-off process with two-layer photoresists. The SiO_2_ cladding layer was deposited by plasma-enhanced chemical vapour deposition and polished by chemical-mechanical polishing, leaving a 100-nm overcladding above the SiN waveguide. In the central area of the chip for the coplanar waveguide, RIE recessed the oxide layer until the underlying Cr was exposed, then continued for an additional 600 nm so that the gold layer lay below the top silicon dioxide surface, enabling direct bonding with the TFLN. The electrode area was patterned by the stepper, and then 900 nm gold was deposited by an electron-beam evaporator tool, followed by metal lift-off process. A 100-mm-diameter, 300-nm lithium niobate-on-insulator (LNOI) wafer was thinned by chemical-mechanical polishing to a final thickness of 150 nm. Both wafers (SiN and LNOI) were coated with 5 nm of Al_2_O_3_ via atomic layer deposition. The diced LNOI piece was flip-chip-bonded onto the SiN wafer at room temperature and atmospheric pressure. The bonded wafer was annealed at 200 °C for 1 h to increase the bonding strength. The wafer was diced into chips, and their edges were polished using a diamond polish pad.

### Insertion loss

The fibre-to-chip coupling loss is measured at 5.35 dB ± 0.30 dB per facet. The excess loss comes from the limited resolution of the 365-nm photolithography tool, which cannot define the inverse taper tip narrowly enough. Device insertion loss is measured at 8.05 dB ± 0.28 dB at a nominal wavelength of 800 nm. We attribute the excessive insertion loss to imperfect fabrication during the bonding of the buried electrodes. Because this bonding process is not yet optimized, it may introduce additional scattering and increased optical propagation loss. However, the isolation principle can be applied to any TW modulator with low optical propagation loss. Assuming a perfect bonding process, the total insertion loss of the device would be dominated by the passive components. Importantly, in the ideal case where passive losses such as the insertion loss of the directional couplers and the waveguide propagation loss are minimized, the DRDI itself introduces zero intrinsic insertion loss in the forward direction.

### PIC mode management

To minimize mode mismatch loss between the SiN-only waveguide and the SiN/TFLN hybrid waveguide, which arises from the higher refractive index of the TFLN, the SiN waveguide width is expanded to ~2 μm near the edge of the bonding interface, confining the optical mode entirely within SiN and suppressing its evanescent overlap with TFLN. After the edge of bonding interface, the SiN width is adiabatically reduced to nominal 700 nm, transferring the mode into the TFLN for efficient electro-optic modulation. At the other end of the bonding interface, the SiN width is back to ~2 μm for the same strategy. The redundant unused output port of the 2-by-2 directional coupler is routed adjacent to the buried gold electrode, which absorbs the residual light and prevents back-reflection.

### Data analysis

Wherever unspecified in the text, reported uncertainties are 68% confidence intervals, corresponding to one standard deviation statistical uncertainty of the mean. The statistical uncertainties are calculated from five repeated measurements.

## Online content

Any methods, additional references, Nature Portfolio reporting summaries, source data, extended data, supplementary information, acknowledgements, peer review information; details of author contributions and competing interests; and statements of data and code availability are available at https://doi.org/10.1038/s41566-026-01978-0.

## Supplementary information


Supplementary InformationSupplementary Sections 1–5, Supplementary Figs. 1–6 and captions for Supplementary Videos 1 and 2.
Peer Review File
Supplementary Video 1Animated version of the analysis shown in Fig. 2c–e for the DRDI scheme with the triangular waveform. The video shows the accumulated phases of the four channels over one RF period, the corresponding time-segmented unwrapped phases and the phasor-space dynamics of the rotating cancellation basis. Two channel pairs remain separated by π in phase, producing continuous destructive interference of the backward-propagating light.
Supplementary Video 2Animated DRDI analysis for the theoretically optimized waveform with *N* = 3 as shown in Fig. 3a. The video shows the accumulated phases, time-segmented unwrapped phases and phasor-space dynamics in the same format as in Fig. 2c–e. The rounded peaks, dips and zero-crossings preserve the rotating cancellation condition while removing the derivative discontinuities of the triangular-wave case.


## Data Availability

The data that support the findings of this study are available at https://doi.org/10.18434/mds2-4202.

## Extended data

**Extended Data Table 1:** Comparison of on-chip integrated optical isolators

Material	Isolation Mechanism	Device Structure	Isolation^‡^ (dB)	Bandwidth^†^ (THz)	Wavelength (nm)	Insertion Loss* (dB)	Ref
SiN/TFLN	EO/Spatiotemporal	MZM	30	4.8	790	8.05	This work
Si	TO	μ-ring resonator	28	< 0.01	1630	12	^43^
Si	Plasma/Temporal	μ-ring	13.1	N/A	1555	18.1	^22^
Si	Plasma/Mode coupling	Slotted waveguide	3	N/A	1558	70	^17^
Si	Plasma/Mode coupling	Slotted waveguide	2.4	N/A	1570	Not explicitly reported	^18^
Si	AO/Mode coupling	Two multimode waveguides	38	0.13	1548	20**	^30^
AlN	AO/Mode coupling	Racetrack resonator	15	N/A	1542	Not explicitly reported	^31^
Si/AlN	AO/Mode coupling	Single multimode waveguide	16	N/A	1524	> 6**	^27^
TFLN	AO/Mode coupling	Racetrack resonator	39.3	< 0.01	1526	0.65	^28^
SiN/AlN	AO/Mode coupling	μ-ring resonator	10	N/A	1546	< 1	^29^
Si/AlN	AO/Mode coupling	Multi-mode interferometer	24.5	≈ 0.8	1560	8.09	^32^
Si/Ce:YIG	MO/non-symmetric tensor	Racetrack resonator	19.5	N/A	1542	18.8	^13^
Si/Ce:YIG	MO/non-symmetric tensor	μ-ring resonator	32	< 0.02	1552	2.3	^11^
Si/Ce:YIG	MO/non-symmetric tensor	MZM	29	2.3	1523	9	^9^
Si/Ce:YIG	MO/non-symmetric tensor	Adiabatic taper	16	N/A	1561	13	^10^
SiN/Ce:YIG	MO/non-symmetric tensor	MZM	28	3.5	1588	2.7	^14^
TFLN	Nonlinear/χ^(2)	Single waveguide	18	N/A	1565	1	^44^
SiN	Nonlinear/χ^(3)	μ-ring resonator	35	< 0.01	1550	5.5	^45^
SiN	Nonlinear/χ^(3)	μ-ring resonator	14	N/A	1550	Not explicitly reported	^46^
InP	EO/Spatiotemporal	Single waveguide	< 6	N/A	1580	2.3	^21^
Bulk LN	EO/Spatiotemporal	MZM	12.5	N/A	1552	5.5	^25^
TFLN	EO/Spatiotemporal	Single waveguide + μ-ring	34	< 0.01 (filter linewidth)	1552	0.5	^33^
TFLN	EO/Temporal	Single waveguide + μ-ring	< 10	N/A	775, 1550	< 0.5	^26^
GaAs–AlGaAs	EO/Spatiotemporal	MZM	30	3.2	1550	<23.8***	^20^

^‡^Peak isolation (dB) = 10 log10(T_fwd/T_bwd)

^†^Bandwidth for isolation > 20 dB without active tuning

*Unless noted, insertion loss excludes fiber-to-chip coupling loss.

**Maximum modulation efficiency to sideband

***The paper does not explicitly report fiber-to-chip coupling loss; therefore the value is the total insertion loss.

Forward-propagating light is modulated by single-sideband modulation, which causes fundamentally unavoidable 6 dB loss and undesired frequency shift.

AO: acousto-optic effect; TO: thermo-optic effect; EO: electro-optic effect; MO: magneto-optic effect; Plasma: plasma dispersion effect.
