# Supplementary material for: Integrated optical isolators for broadband multi-laser operation
Source: Nat Photonics. 2026 Aug 13;20(9):1107–15. doi: 10.1038/s41566-026-01978-0 (PMC13533830; doi:10.1038/s41566-026-01978-0)
Supplement: Supplementary file 1 [file 41566_2026_1978_MOESM1_ESM.pdf]

nature photonics

Supplementary information

https://doi.org/10.1038/s41566-026-01978-0

Integrated optical isolators for broadband multi-laser operation

In the format provided by the authors and unedited

SUPPLEMENTARY INFORMATION

Table of Contents

Supplementary Section 1. Determination of the base RF frequency for maximizing forward transmission 2

Supplementary Section 2. RF and heater optimization 2

Supplementary Section 3. RF waveform requirement for DRDI 4

Supplementary Section 4. Theoretical model using the transfer matrix analysis 6

Supplementary Section 5. Captions for Supplementary Videos 8

Supplementary Section 1. Determination of the base RF frequency for maximizing forward transmission

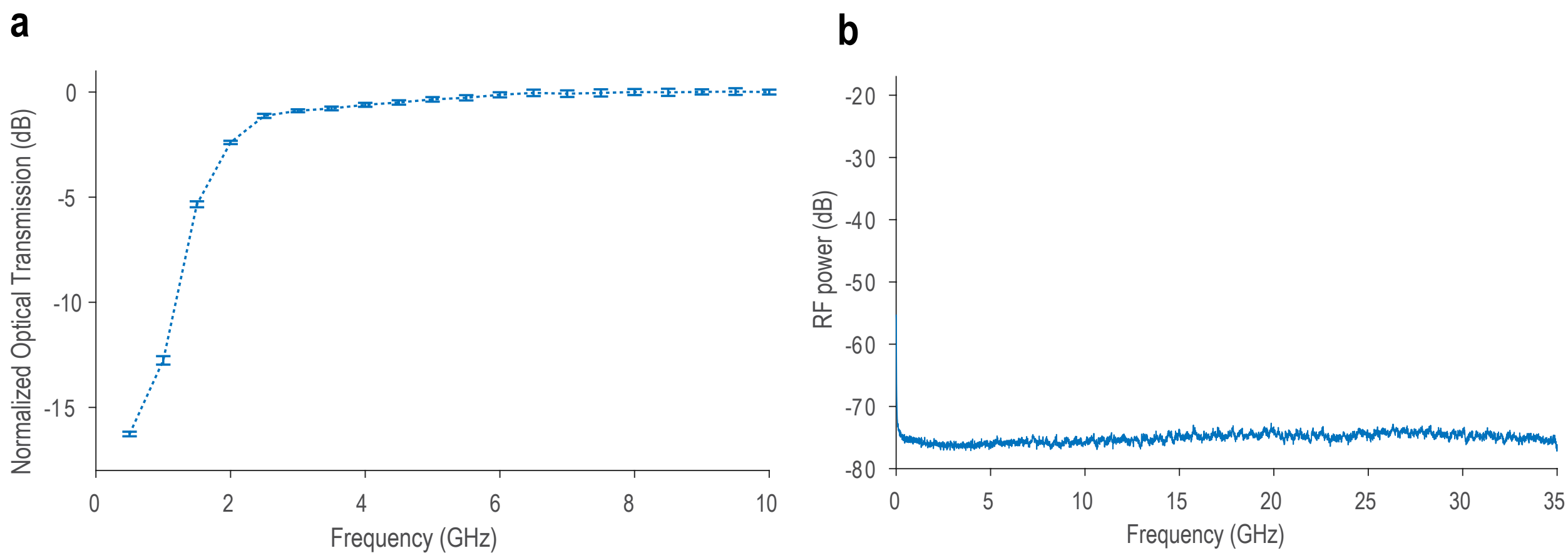

Figure S1. Frequency response of the forward propagating light **a**. Normalized optical transmission as a function of sinusoidal RF drive frequency. At 0.5 GHz, the transmission is strongly attenuated below -15 dB. As frequency increases, the zero mean phase modulation yields a reduced net phase shift and higher optical transmission. All values are normalized to the transmission at 10 GHz. The plotted dashed lines connect the average normalized optical transmission values obtained from five RF frequency-sweep measurements on the same isolator device. At each frequency, one normalized-transmission value was extracted from each sweep. The error bars indicate ± one-standard-deviation statistical uncertainty of the mean normalized transmission. **b**. RF power spectrum of the forward-propagating light. Measured resolution bandwidth is 5 MHz. The RF power scale is referenced to 1 mW.

We swept the sinusoidal RF drive frequency from 0.5 GHz to 10 GHz while monitoring the forward propagating optical transmission. Figure S1a shows transmission is strongly attenuated at 0.5 GHz, indicating that the electro-optic modulator remains in a near electrostatic regime in which the device lengths are much shorter than the wavelength of the RF wave. As frequency increases, the net accumulated phase shift of the forward propagating light decreases because the periodic RF waveform has zero mean, leading to increased transmission. Above approximately 2.5 GHz, the slope begins to flatten, and the response saturates around 6.5 GHz. We therefore selected 6.5 GHz as the base RF frequency for driving, since higher frequencies give no further improvement in forward transmission. Figure S1b presents the RF spectrum of the forward propagating optical signal at a 6.5 GHz drive frequency, measured with a high frequency photoreceiver. The DC power level was approximately 32 µW, and the residual phase modulation sidebands stay below noise level.

Supplementary Section 2. RF and heater optimization

As shown in Figure S2, the experimental setup comprises an external-cavity diode laser followed by an in-line optical fiber polarization controller, which sets the light to transverse-electric (TE) polarization at the isolator chip. Multi-contact RF probes, each carrying two RF and six DC lines, are placed at the CPW input and output. At the CPW output, the CPW may be terminated with a 50 Ω load or connected to an RF spectrum analyzer (also 50 Ω), giving identical impedance conditions. An arbitrary waveform generator (AWG) with two independent RF channels produces multi-tone waveforms, which are amplified by RF amplifiers. Two three-channel DC power supplies drive the on-chip microheaters. The isolator's output light is split via 50:50 directional couplers into three paths. An optical power meter measures time-averaged optical power. An optical spectrum analyzer detects any high-order harmonics. A high-frequency photoreceiver measures residual phase-modulations. All instruments are controlled by, and their data are acquired on a workstation.

The microheaters in the isolator chip are driven by two DC power supplies controlled by a workstation. Because the split ratio of the parallel-waveguide directional couplers depends on wavelength, each operating wavelength requires its own optimized set of DC powers. As described in Figure S3, we use an optical microscope to observe

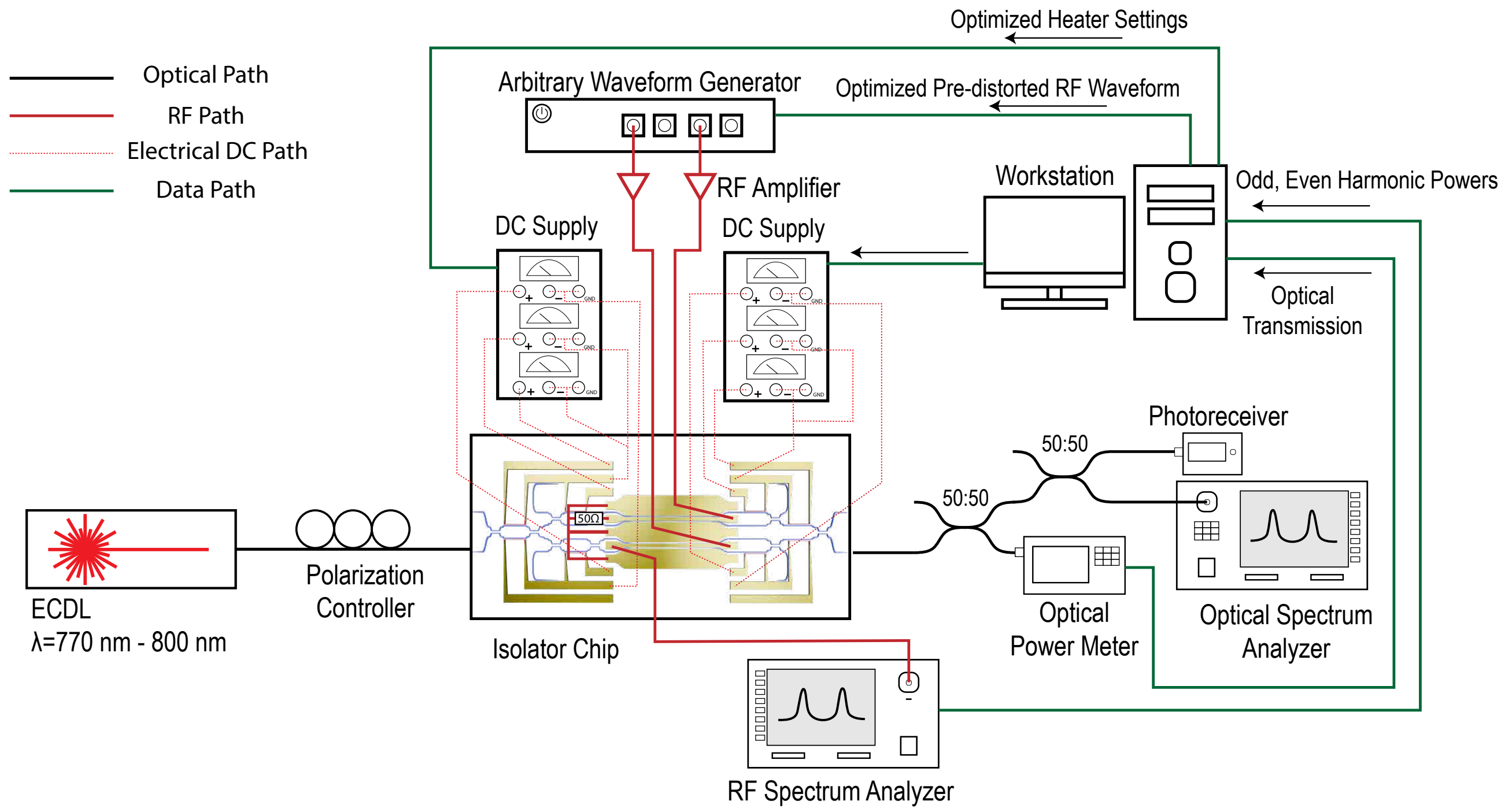

Figure S2. Experimental setup for the DRDI isolator characterization. The external-cavity diode laser is polarization-controlled and coupled into the isolator chip via tapered lensed fiber with a nominal mode field diameter of 2 µm. Multi-contact RF probes deliver two RF lines (solid red) to the CPW and six DC lines (dashed red) to on-chip microheaters. A two-channel AWG feeds pre-distorted RF wave. All instruments connect via data/control lines (green) to a central workstation, which iteratively adjusts the AWG waveform and heater settings.

scattered light and adjust Heater 1 (H1) to route all input laser power into either the upper push-pull pair (Ch 1 and 2) or the lower push-pull pair (Ch 3 and 4). With H1 fixed for the upper pair, we sweep heater 4 (H4) and measure the output optical power. The transmission minimum marks the out-of-phase condition, and the maximum marks the in-phase condition. The out-of-phase point is easier to identify since its transmission change exceeds noise fluctuations. To locate the in-phase point accurately, we fit the transmission data to a sinusoidal function to suppress noise. Next, holding H4 at its out-of-phase setting, we sweep heater 2 (H2) to balance power between Ch1 and Ch2, again indicated by a transmission minimum. We then set H1 to direct most power into the lower pair and repeat the optimization for that pair by sweeping heater 5 (H5) and 3 (H3) to minimize transmission. After both pairs are optimized, we set H4 and H5 to their in-phase conditions to establish the dynamic rotating destructive interference (DRDI). Finally, we sweep heater 6 (H6) to find its out-of-phase point, use that to fine-tune H1 for equal splitting between the two pairs (minimum transmission), and then set H6 to its in-phase point for the final DRDI operation.

The optimized heater powers at each wavelength change almost linearly across the wavelength range from 770 nm to 800 nm as shown in Figure S4. The discrete steps on H6 arise from the coarse scanning increments used in the optimization for faster optimization. Importantly, the heater settings vary continuously without jumps, enabling faster and more stable wavelength sweeps. This can be advantageous for any spectroscopic applications with sweeping ECDL wavelength. We confirmed that measuring only a few wavelengths and interpolating the heater powers between them gives nearly identical isolation to the method of optimizing heaters at every wavelength.

Because of nonlinearity, frequency-dependent phase delay, and attenuation, we must optimize the RF drive waveform to maximize isolation. We generate a multi-tone waveform that its degree of freedom are the magnitudes and phases of the fundamental and its odd harmonics. Truncating the harmonics to only odd harmonics simplifies the optimization by eliminating redundant parameters required by the DRDI scheme. The detailed procedure is shown in Figure S5. First, the AWG outputs the theoretically predicted waveform, and we compare the measured RF spectrum at the CPW output with the ideal spectrum to adjust the relative powers of the high-

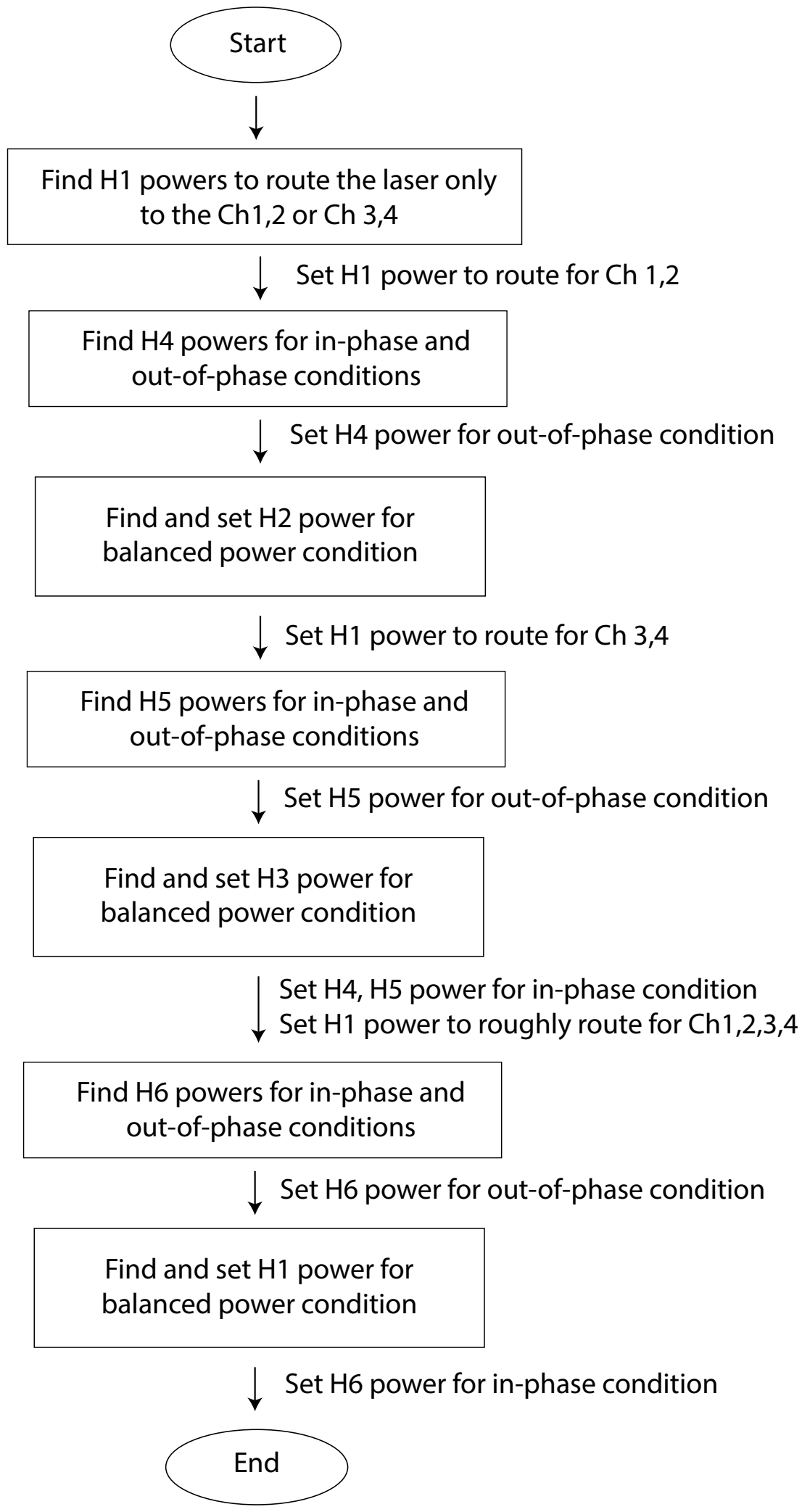

Figure S3. Flowchart for the microheater optimization. H1 first routes light into either the upper or lower push-pull pair. Each pair is set at the out-of-phase condition, then adjusted to balance power, and then switched to the in-phase condition. H6 is tuned to the out-of-phase condition to balance power between the pairs, then switched to the in-phase condition.

order odd harmonics. Next, for each push-pull pair, we sweep the AWG output power to identify the setting that minimizes the backward optical transmission. We then optimize each odd harmonic by adjusting their phases and magnitudes individually. Because even harmonics are unnecessary for DRDI, we adjust their phases and amplitudes to compensate for any unwanted even harmonic generation by the RF amplifiers. We repeat the entire cycle until the target isolation is reached.

Supplementary Section 3. RF waveform requirement for DRDI

Although a triangular waveform is a valid solution for DRDI, it is impractical because truncating its harmonics dramatically degrades isolation. To find an optimal waveform within a given RF bandwidth, we generalize the required RF waveform described in Figure S6a. First, the RF waveform must be periodic and zero-mean, so that the forward propagating optical wave experiences no net phase shift in the traveling-wave modulator. For destructive interference between four optical waves driven by two RF signals delayed by one-quarter RF period,

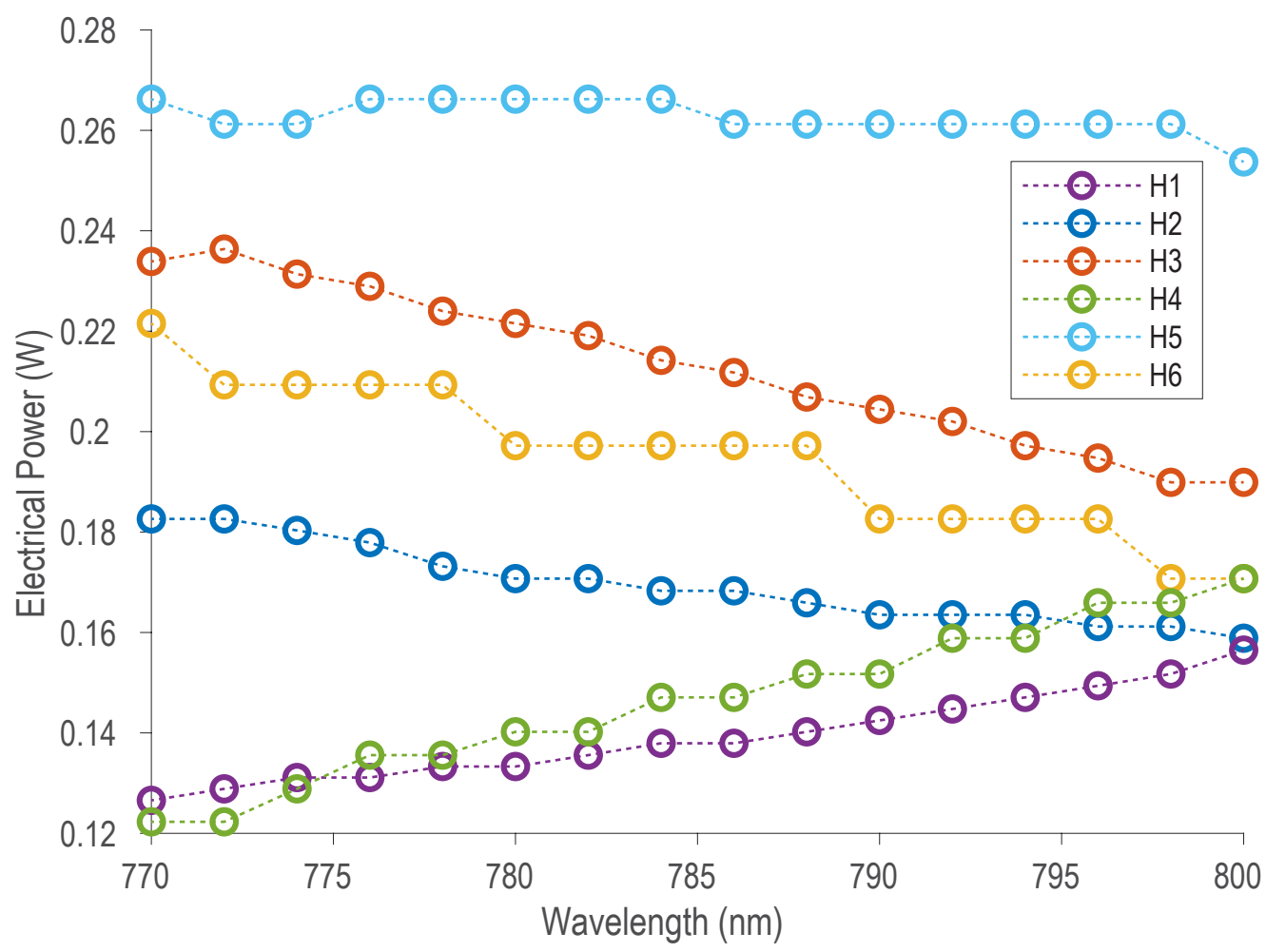

Figure S4. Optimized heater powers for various wavelengths. Electrical powers for H1-H6 are plotted as a function of the laser wavelengths. All heaters exhibit linear tuning across the wavelength range.

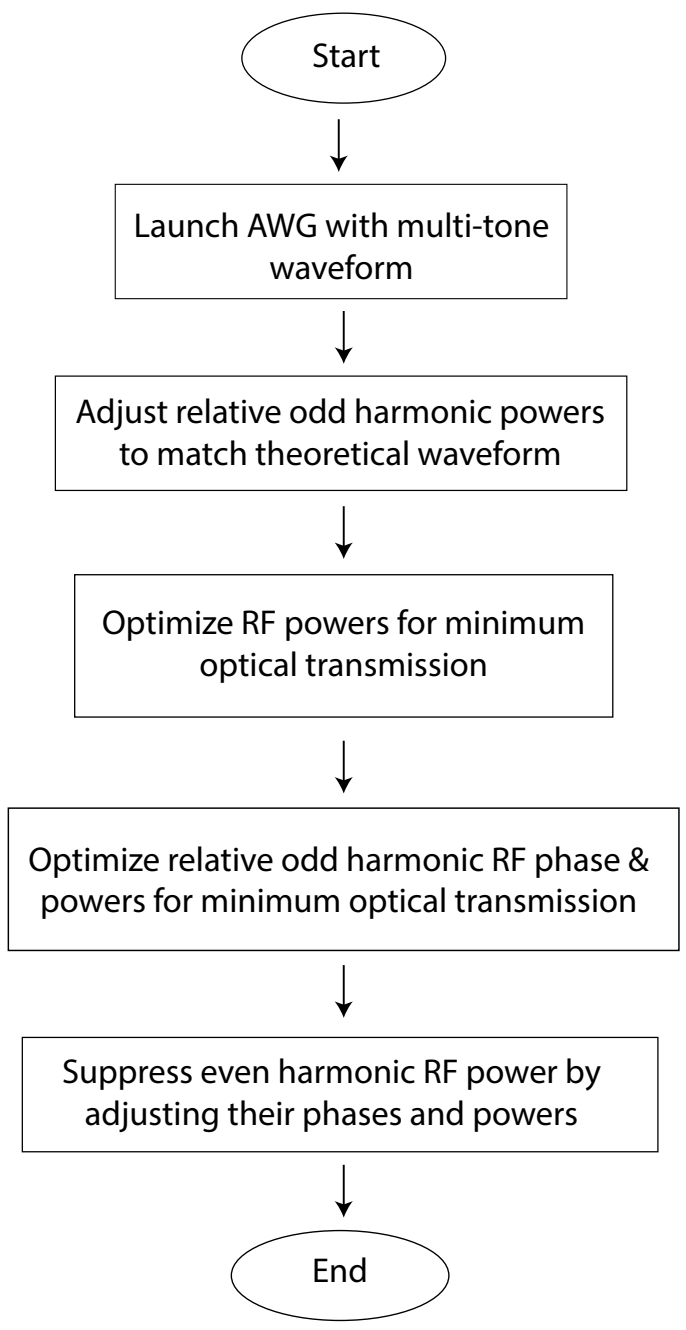

Figure S5. Flow chart for RF waveform optimization. To overcome nonlinearity and attenuation in the RF path, we generated a multi-tone RF waveform consisting of the fundamental and odd harmonics and iteratively adjusted their amplitudes and phases based on the measured CPW output spectrum and optical transmission minima. Any residual even harmonics are suppressed by fine tuning their phases and powers.

we begin with a base RF voltage waveform $u(t)$ defined on the quarter-period interval from $T_0/4$ to $T_0/2$. The boundary conditions of the base waveform are

$$u\big(\tfrac{T_0}{4}\big) = 0, \quad u\big(\tfrac{T_0}{2}\big) = V_\pi \tag{S1}$$

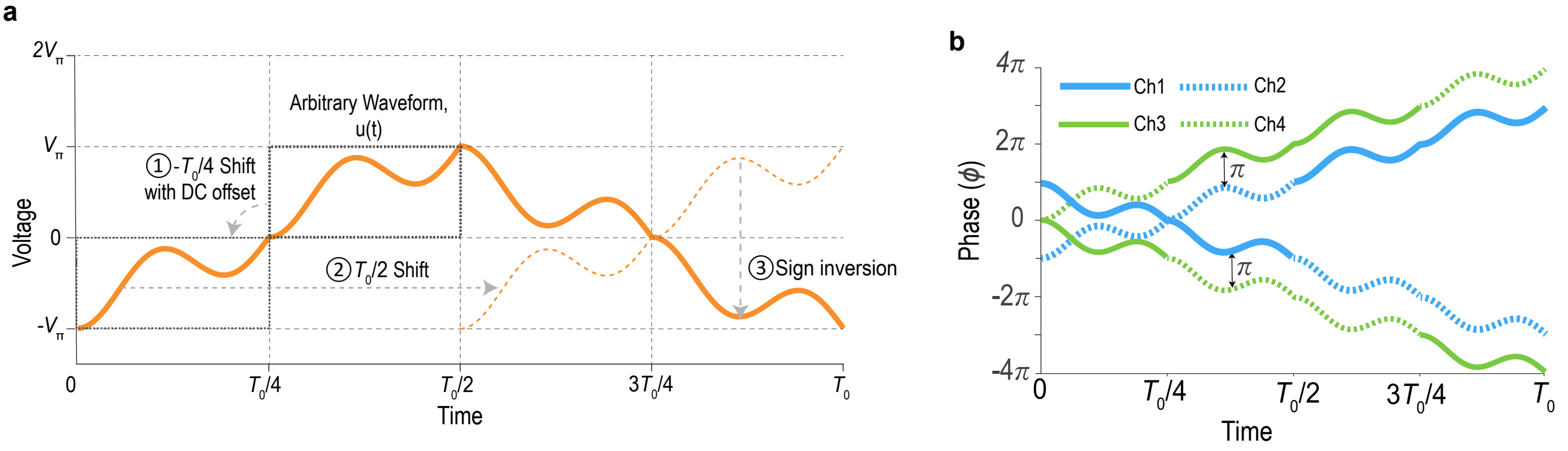

Figure S6. Generalized RF waveform synthesis and resulting phases for the DRDI. **a**. Arbitrary base waveform $u(t)$ defined on the quarter-period interval is extended over the full period. The solid line shows the resulting period, zero-mean waveform enforcing half-wave symmetry **b**. Time-segmented, unwrapped accumulated phases of the four channels over one RF period (T_0). At each quarter-period, the channels form two cancellation pairs, each maintaining a π phase difference, implementing the DRDI scheme.

Then we can extend the RF voltage waveform $f(t)$ to all t by enforcing periodicity $f(t-T_0) = f(t)$ and half-wave symmetry $f(t - T_0/2) = -f(t)$. This can be achieved for the period $0 \le t \le T_0$ with the conditions

$$f(t) = \begin{cases} u(t + T_0/4) - V_\pi, & 0 \le t < T_0/4, \\ u(t), & T_0/4 \le t < T_0/2, \\ -f\big(t - T_0/2\big), & T_0/2 \le t < T_0. \end{cases} \tag{S2}$$

Because of Equation (S1), these conditions guarantee continuity at the quarter-period boundaries, while automatically satisfying half-wave symmetry. The resulting RF drive waveform satisfies the DRDI condition, ensuring that the phase-modulated four optical channels destructively interfere at the output waveguide at all times, as shown in Figure S6b.

Supplementary Section 4. Theoretical model using the transfer matrix analysis

To mathematically model the dynamic response of our device in the time domain, we employ the transfer matrix analysis. The fundamental building block is the ideal 2×2 directional coupler with 50:50 split, which is represented by the transfer matrix T_c

$$T_c = \frac{1}{\sqrt{2}} \begin{pmatrix} 1 & i \\ i & 1 \end{pmatrix} \tag{S3}$$

As illustrated in the schematics in the main text, the 1×4 splitting section consists of cascaded directional couplers. For simplicity, however, we model only the power-control section as an idealized three-stage MZI tree that generates four equal-amplitude channels. Although this simplified model does not capture the full physical implementation of the chip, it is sufficient to demonstrate that DRDI can maintain complete destructive interference at all times. Let the state of the complex optical electric field in the four input and output waveguide ports (ordered from top to bottom: 3, 2, 1, 4) be represented by a column vector. The input light enters through the port 1, giving the initial state vector $E_{in} = (0, 0, 1, 0)^T$.

The splitting process occurs in two stages. The first stage (M_1) couples the inner waveguides (ports 1 and 2), and the second stage (M_2) consists of two parallel couplers mixing the outer and inner waveguides. The transfer

matrices for these stages are

$$M_1 = \begin{pmatrix} 1 & 0 & 0 & 0 \\ 0 & \frac{1}{\sqrt{2}} & \frac{i}{\sqrt{2}} & 0 \\ 0 & \frac{i}{\sqrt{2}} & \frac{1}{\sqrt{2}} & 0 \\ 0 & 0 & 0 & 1 \end{pmatrix}, \quad M_2 = \frac{1}{\sqrt{2}} \begin{pmatrix} 1 & i & 0 & 0 \\ i & 1 & 0 & 0 \\ 0 & 0 & 1 & i \\ 0 & 0 & i & 1 \end{pmatrix} \tag{S4}$$

The optical electric field distribution entering the phase modulators is obtained by multiplying these matrices with the input vector

$$V_{split} = M_2 M_1 E_{in} = \begin{pmatrix} -1/2 \\ i/2 \\ 1/2 \\ i/2 \end{pmatrix} \tag{S5}$$

The four electro-optic phase modulators introduce time-varying phase shifts with phase offsets by the heaters (or passive optical delay lines), which can be represented by a diagonal matrix $M_\phi(t)$ with the optical channel numbers as specified in Figure 2b. Finally, the output combiner section is structurally symmetric to the splitter. Therefore, the total complex transmission at the port 1, $\tau(t)$, is given by projecting the combined field

$$\tau(t) = \begin{pmatrix} -1/2 & i/2 & 1/2 & i/2 \end{pmatrix} \begin{pmatrix} e^{i(\phi_1(t)+\theta_1)} & 0 & 0 & 0 \\ 0 & e^{i(\phi_2(t)+\theta_2)} & 0 & 0 \\ 0 & 0 & e^{i(\phi_3(t)+\theta_3)} & 0 \\ 0 & 0 & 0 & e^{i(\phi_4(t)+\theta_4)} \end{pmatrix} \begin{pmatrix} -1/2 \\ i/2 \\ 1/2 \\ i/2 \end{pmatrix} \tag{S6}$$

$$\tau(t) = \frac{1}{4} \left\{ e^{i(\phi_1(t)+\theta_1)} - e^{i(\phi_2(t)+\theta_2)} + e^{i(\phi_3(t)+\theta_3)} - e^{i(\phi_4(t)+\theta_4)} \right\} \tag{S7}$$

The negative signs in Equation (S7) represent the intrinsic π phase shifts introduced by the cross-coupling terms of the cascaded directional couplers. To ensure perfect destructive interference driven strictly by the RF waveforms, we bias the device to an in-phase condition by applying appropriate static phase offsets via the microheaters. Alternatively, this static phase compensation can be achieved passively by designing the waveguides with asymmetric lengths. Specifically, by setting $\theta_1 = 0$, $\theta_2 = \pi$, $\theta_3 = 0$, and $\theta_4 = \pi$, we can achieve in-phase condition between the four optical channels.

$$\tau(t) = \frac{1}{4} \left\{ e^{i\phi_1(t)} + e^{i\phi_2(t)} + e^{i\phi_3(t)} + e^{i\phi_4(t)} \right\} \tag{S8}$$

Furthermore, our device operates in a push-pull configuration, where the respective channels of the dual-drive Mach-Zehnder modulators provide equal and opposite phase shifts. This dictates that $\phi_2(t) = -\phi_1(t)$ and $\phi_4(t) = -\phi_3(t)$ for all times t. Substituting these conditions into Equation (S8) yields

$$\tau(t) = \frac{1}{4} \left\{ e^{i\phi_1(t)} + e^{-i\phi_1(t)} + e^{i\phi_3(t)} + e^{-i\phi_3(t)} \right\} \tag{S9}$$

Using Euler's formula, this equation simplifies to real-valued cosine functions

$$\tau(t) = \frac{1}{2} \left\{ \cos(\phi_1(t)) + \cos(\phi_3(t)) \right\} \tag{S10}$$

Based on Equation (S10), we can explicitly verify the perfect destructive interference at the specific time instances highlighted in Figure 2e.

First, in the case where Channel 1 and Channel 3 cancel each other out at $t = T_0/32$ and $t = 19T_0/32$, the phase relationship is $\phi_3(t) = \phi_1(t) - \pi$. Substituting this into Equation (S10) yields obvious signal cancellation

$$\tau(t) = \frac{1}{2} \left\{ \cos(\phi_1(t)) + \cos(\phi_1(t) - \pi) \right\} = \frac{1}{2} \left\{ \cos(\phi_1(t)) - \cos(\phi_1(t)) \right\} = 0 \tag{S11}$$

Second, in the case where Channel 1 and Channel 4 cancel each other out at $t = 10T_0/32$ and $t = 26T_0/32$, the required phase relationship is $\phi_4(t) = \phi_1(t) - \pi$. Given the intrinsic push-pull condition of the device ($\phi_4(t) = -\phi_3(t)$), this relation is mathematically equivalent to $\phi_3(t) = -\phi_1(t) + \pi$. Substituting this condition into Equation (S10) also yields perfect cancellation

$$\tau(t) = \frac{1}{2}\left\{\cos(\phi_1(t)) + \cos(-\phi_1(t) + \pi)\right\} = \frac{1}{2}\left\{\cos(\phi_1(t)) - \cos(\phi_1(t))\right\} = 0 \tag{S12}$$

These derivations mathematically confirm that our phase-compensated theoretical model completely guarantees $\tau(t) = 0$ in both cancellation scenarios, supporting the complete theoretical isolation of our work.

Supplementary Section 5. Captions for Supplementary Videos

Supplementary Video 1. Animated version of the analysis shown in Fig. 2c–e for the DRDI scheme with the triangular waveform. The video shows the accumulated phases of the four channels over one RF period, the corresponding time-segmented unwrapped phases, and the phasor-space dynamics of the rotating cancellation basis. Two channel pairs remain separated by π in phase, producing continuous destructive interference of the backward-propagating light.

Supplementary Video 2. Animated DRDI analysis for the theoretically optimized waveform with $N = 3$ as shown in Fig. 3a. The video shows the accumulated phases, time-segmented unwrapped phases, and phasor-space dynamics in the same format as Fig. 2c–e. The rounded peaks, dips, and zero-crossings preserve the rotating cancellation condition while removing the derivative discontinuities of the triangular-wave case.
