# Supplementary material for: Integrated optical isolators for broadband multi-laser operation
Source: Nat Photonics. 2026 Aug 13;20(9):1107–15. doi: 10.1038/s41566-026-01978-0 (PMC13533830; doi:10.1038/s41566-026-01978-0)
Supplement: Supplementary file 2 [file 41566_2026_1978_MOESM2_ESM.pdf]

nature portfolio

Peer Review File

Integrated optical isolators for broadband multi-laser operation

Corresponding Author: Dr Vladimir Aksyuk

This manuscript has been previously reviewed at another journal. This document only contains information relating to versions considered at Nature Photonics.

A version of this paper was originally rejected for publication by Nature Photonics, however that decision was reconsidered after appeal by the authors.

Version 1:

Reviewer comments:

Reviewer #1

(Remarks to the Author)
In the presented paper, the authors have experimentally demonstrated an optical isolator based on dynamic modulation of the waveguides reaching the isolation depth of >24 dB across a 30-nm wavelength range with its insertion loss of approximately 8 dB. Previous demonstrations of the optical isolator based on the dynamic modulation had limitations such as narrow transmission bandwidth, low isolation or significant insertion loss. The achievement of realizing an order broader wavelength range, as compared to the previous demonstrations of non-magnetic designs, is based on the dynamic rotating destructive interference (DRDI) method established in this paper. The realization of optical isolator based on foundry-ready process would be of wide interest to the readers. The paper is well constructed and sufficient details are provided. I would like to suggest publication of this paper if the following comments are addressed.
1. The fabricated device is based on SiN waveguides integrated with flip chip bonded thin film lithium niobate (TFLN). Although mass fabrication technologies of TFLN devices are gaining much attention, could the authors discuss prospects toward the foundry compatibility? This discussion would be important as it is related to the title of the paper presented.
2. Could the authors comment on the power consumption of the device with the breakdown of the power consumption by components? Although it is shown that the RF power output from the CPW was 64 mW, it would be helpful if the authors could discuss the RF loss of the system to show the actual power output from the RF amplifier.
3. Because the proposed method uses optical interference, some readers might be interested in temperature tolerance of the device. Could the authors comment on the tolerance of the device against the temperature variations?
4. Could the authors comment on the possible spectral degradation of the light through the chip due to the dynamic modulation, which might be related to some atomic optical applications such as atomic clocks?

Reviewer #2

(Remarks to the Author)
This work demonstrates an on-chip optically broadband isolator with several practical advantages over other common techniques. Most notably, they demonstrate simultaneous isolation of two separate lasers tuned to wavelengths several nm apart. This is impressive and I believe it lays the groundwork for a standard approach to optical isolation on material platforms where electro-optics are possible.

Following are some comments to the authors:
1. page 2 left column, second para: sentence starts with "Previous work..." Can the authors add the reference number after this sentence?
2. Figure 1 on page3: It is a bit difficult to understand the hybrid mode structure between the SiN and LN wave-guides based on the main text of the paper alone (despite being present in the supplement/extension). A false-color SEM or simulated hybrid mode with materials clearly labeled should be presented as part of this figure for clarity.
3. Page 7 bottom left...section about "to maximize isolation".
Can the authors comment on whether or not they believe the RF waveform the found is unique given bandwidth constraints (i.e., some global optimal)?

4. Page 7 bottom left...section about "to maximize isolation". (this is a suggestion and I defer how/if this should be addressed to the authors and the editor): The requirement of specific harmonics potentially precludes platforms without electro-optics from benefiting from the techniques outlined in this work. I think it would add a large amount of value to the paper to analyze the case where driving at an exact harmonic is not possible in the SI. A simple case to analyze could be using your optimized waveform in simulation and changing the 3rd harmonic to e.g. the "2.8-th" harmonic instead and seeing what the predicted isolation contrast becomes. This analysis would help generalize this technique to other platforms where acousto-optics is the preferred modulations strategy.
5. page 12 discussion on Insertion Loss: if the bonding process were to be perfected, what insertion loss do the authors believe would be achievable?
Two questions involve equations and are provided in pdf and txt format.

Professor Sunil Bhave and Graduate Student Researcher Scott Kenning

Reviewer #3

(Remarks to the Author)
This manuscript reports a magnet-free integrated optical isolator based on a four-channel electro-optic traveling-wave architecture implementing dynamic rotating destructive interference (DRDI). The device is realized on a SiN/TFLN platform and demonstrates about 30 dB isolation near 789.7 nm, more than 24 dB isolation across a 30 nm span with heater-assisted tuning, and simultaneous more than 20 dB isolation for two lasers across a 10 nm range. Overall, I find the work novel, significant, and potentially suitable for publication in Nature Photonics.

The paper addresses an important long-standing challenge in integrated photonics: achieving broadband non-magnetic on-chip isolation without magneto-optic materials. The DRDI concept is elegant and well explained, and the experimental results are compelling. I also view the dual-laser experiment as an important strength, because it moves the paper beyond a narrow single-wavelength proof of concept and supports the broader relevance of the approach.

I have only a few suggestions to strengthen the manuscript further. First, the broadband claim should be phrased more carefully, since the best 30 nm result relies on active heater tuning at different wavelengths; this should be distinguished more clearly from fixed-bias operation. Second, the forward-transmission advantage should be quantified more clearly in the main text, since this is one of the key conceptual benefits of the approach. The manuscript states that the forward spectrum under RF is almost identical to the RF-off case, indicating no added loss or frequency shift, and this point deserves a more explicit quantitative presentation.

I also suggest updating the comparison table to include the recent Nature Photonics demonstration of an integrated terahertz-bandwidth non-magnetic isolator (https://www.nature.com/articles/s41566-025-01663-8). That work is directly relevant to the present manuscript and would provide a useful recent benchmark for bandwidth, insertion loss, and mechanism-level comparison.

In summary, this is a strong and original manuscript that makes an important contribution to integrated nonreciprocal photonics. I am supportive of publication in Nature Photonics after minor revision.

Version 2:

Reviewer comments:

Reviewer #1

(Remarks to the Author)
The authors have answered all questions and comments from the Reviewers. The quality of the paper after the revision has been much improved. I would recommend publication of the manuscript in Nature Photonics, and I have no further questions for the authors.

Reviewer #2

(Remarks to the Author)
The Authors' response and updates to the manuscript have addressed all our suggestions. We recommend the manuscript for publication.

Sunil Bhave and Scott Kenning (Graduate Student)

Reviewer #3

(Remarks to the Author)
I have checked the revised manuscript and response, I believe it’s good for publication.

Open Access This Peer Review File is licensed under a Creative Commons Attribution 4.0 International License, which permits use, sharing, adaptation, distribution and reproduction in any medium or format, as long as you give appropriate credit to the original author(s) and the source, provide a link to the Creative Commons license, and indicate if changes were made.
In cases where reviewers are anonymous, credit should be given to 'Anonymous Referee' and the source.
The images or other third party material in this Peer Review File are included in the article's Creative Commons license, unless indicated otherwise in a credit line to the material. If material is not included in the article's Creative Commons license and your intended use is not permitted by statutory regulation or exceeds the permitted use, you will need to obtain permission directly from the copyright holder.
To view a copy of this license, visit https://creativecommons.org/licenses/by/4.0/

Reviewer #1 :

In the presented paper, the authors have experimentally demonstrated an optical isolator based on dynamic modulation of the waveguides reaching the isolation depth of >24 dB across a 30-nm wavelength range with its insertion loss of approximately 8 dB. Previous demonstrations of the optical isolator based on the dynamic modulation had limitations such as narrow transmission bandwidth, low isolation or significant insertion loss. The achievement of realizing an order broader wavelength range, as compared to the previous demonstrations of non-magnetic designs, is based on the dynamic rotating destructive interference (DRDI) method established in this paper. The realization of optical isolator based on foundry-ready process would be of wide interest to the readers. The paper is well constructed and sufficient details are provided. I would like to suggest publication of this paper if the following comments are addressed.

Author Response: We sincerely thank the reviewer for reviewing our work and for positive and insightful comments, which we have addressed below and have updated the manuscript accordingly.

Reviewer Comment #1: *The fabricated device is based on SiN waveguides integrated with flip chip bonded thin film lithium niobate (TFLN). Although mass fabrication technologies of TFLN devices are gaining much attention, could the authors discuss prospects toward the foundry compatibility? This discussion would be important as it is related to the title of the paper presented.*

Author Response #1: We thank the reviewer for the comment. We agree that discussion about mass-production prospects is crucial for our "foundry-ready" claim. We have added the following discussion to the revised manuscript to clarify the scalability of our heterogeneous integration approach. Additionally, we are aware of at least one commercial foundry (Ligentec) that currently offers a very similar process through a multiproject MPW-LN offering, which may allow implementation of our device.

> The heterogenous integration method we employ for electro-optic modulation is intrinsically suited for mass-production foundries. Alternatives relying on full thin-film lithium niobate (TFLN) wafers as the starting substrate present practical limitations for large-scale production, primarily due to high material costs and the strict constraints on wafer selection limiting co-integration with electronic integrated circuits (EICs). Our PIC platform circumvents these bottlenecks by introducing TFLN strictly during the back-end-of-line (BEOL) compatible bonding phase. In our approach, the underlying SiN PIC can be manufactured in high volume using well-established commercial foundry process flows. From a foundry perspective, handling TFLN solely at the BEOL bonding stage introduces minimal disruption to existing, mature fabrication process flows, providing a practical and flexible route toward mass-production.

Reviewer Comment #2: *Could the authors comment on the power consumption of the device with the breakdown of the power consumption by components? Although it is shown that the RF power output from the CPW was 64 mW, it would be helpful if the authors could discuss the RF loss of the system to show the actual power output from the RF amplifier.*

Author Response #2: Thank you for your suggestion to include this information, which we now provide in the following revision. We agree that it is important for evaluating the practical usage of the isolator.

> To determine the actual RF drive requirements, the average power measured directly after the RF amplifier was 402 mW per channel. After calibrating for RF cable and probe losses, the on-chip RF insertion loss of the isolator was evaluated to be 8.1 dB. Given this propagation loss and the pre-distorted multitone nature of the driving signal, the required on-chip RF voltage naturally exceeds the low-frequency V_π estimation due to the frequency-dependent RF V_π roll-off. In addition to the

RF power, the integrated microheaters consume between 0.12 W and 0.27 W of DC electrical power to balance the optical powers and phase offsets between the channels (Figure S4).

Reviewer Comment #3: *Because the proposed method uses optical interference, some readers might be interested in temperature tolerance of the device. Could the authors comment on the tolerance of the device against the temperature variations?*

Author Response #3: We thank the reviewer for raising this important consideration. As the reviewer pointed out, the thermal dependence of this isolator may be expected to arise primarily from thermal changes in any unbalanced optical path lengths of the individual optical channels. To clarify this thermal behavior for the readers, we have added the following discussion to the revised manuscript.

> While the physical path lengths of the four parallel optical waveguides are designed to be strictly identical, typical fabrication non-idealities such as lithography dose and waveguide layers' thickness variations can introduce slight differences in the effective refractive index of the waveguide modes among the optical channels. These discrepancies can cause the in-phase condition to drift with temperature due to different thermo-optic phase shifts along the modulation region. However, transferring this design to commercial foundry processes with highly calibrated lithography and uniform film deposition will significantly decrease these variations. Furthermore, unlike isolators that rely on narrow-linewidth resonant components such as microrings, our Mach-Zehnder interferometer (MZI)-based design remains intrinsically more robust against temperature-induced performance degradation.

Reviewer Comment #4: *Could the authors comment on the possible spectral degradation of the light through the chip due to the dynamic modulation, which might be related to some atomic optical applications such as atomic clocks?*

Author Response #4: We appreciate the reviewer for this insightful comment. Within the limits of our measurements, no significant spectral degradation due to dynamic modulation is detectable. As shown in Figure S1b, no residual RF tones or noise was detected within the limits of our testing equipment, and Figure 3f confirms the absence of any measurable sidebands in the optical spectra. However, we agree that for applications demanding state-of-the-art stability, such as atomic clocks, a separate detailed high precision and stability study may be needed to evaluate for any excess phase/frequency noise and drift that may arise from the isolator. Unfortunately, such a study is well beyond the scope of the present work. We have added the following discussion to the revised manuscript.

> As demonstrated by our RF and optical spectrum measurements for forward transmission, the DRDI scheme is robust against residual phase modulation, with no observable sidebands or other spectral features generated above the measured noise floor. Therefore, significant degradation of the transmitted light is not expected. However, for applications requiring state-of-the-art frequency stability, such as optical atomic clocks, integrating active feedback loop stabilization may be necessary to further suppress any residual noise.

Reviewer #2 :

This work demonstrates an on-chip optically broadband isolator with several practical advantages over other common techniques. Most notably, they demonstrate simultaneous isolation of two separate lasers tuned to wavelengths several nm apart. This is impressive and I believe it lays the groundwork for a standard approach to optical isolation on material platforms where electro-optics are possible.

Author Response: We sincerely thank the reviewer for the positive evaluation of our work and for the detailed comments that we address below and through the manuscript revisions as follows.

Following are some comments to the authors:
***Reviewer Comment #1.** page 2 left column, second para: sentence starts with "Previous work..." Can the authors add the reference number after this sentence?*

Author Response #1: We thank the reviewer for pointing out this omission. We have added the appropriate references.

***Reviewer Comment #2.** Figure 1 on page3: It is a bit difficult to understand the hybrid mode structure between the SiN and LN wave-guides based on the main text of the paper alone (despite being present in the supplement/extension). A false-color SEM or simulated hybrid mode with materials clearly labeled should be presented as part of this figure for clarity.*

Author Response #2: We fully agree with the reviewer that visualizing the hybrid mode structure directly in the main text is crucial for clarity and readability, and have moved the simulated cross-sectional mode profile (which was previously presented as Extended Data Fig. 4b) into the main text as a new panel in Figure 1f. We have also carefully ensured that the respective materials (SiN and LN) are clearly labeled within the new figure panel.

***Reviewer Comment #3.** Page 7 bottom left...section about "to maximize isolation".*
Can the authors comment on whether or not they believe the RF waveform the found is unique given bandwidth constraints (i.e., some global optimal)?

Author Response #3: We thank the reviewer for raising this highly insightful point. The reviewer is entirely correct that numerical optimization algorithms, such as gradient descent, converge to local optima and in general do not guarantee a unique or global optimum in a multi-dimensional space without additional conditions on the potential and constraints. In our system we start from the physical insight that a perfect triangular wave provides an ideal solution. We then incrementally impose a bandwidth constraint by restricting the number of harmonics and re-optimizing, thereby generating bandwidth-constrained approximations to the triangular waveform that optimize isolation. While it seems likely that the solution obtained in this way may be unique, at least in the vicinity of the minimal necessary RF power, we are unable to provide mathematical proof of this fact. Rather we see the proposed way of generating the waveform as robust and practical way of obtaining a locally-optimal solution satisfying realistic experimental constraints of minimizing bandwidth and harmonic's RF powers. To address the reviewer's comment, we have added the following discussion in the revised manuscript.

> For optimizing the waveform, the parameter space is confined to a small number of amplitudes and relative phases. Given the physical constraints of the electro-optic modulation bandwidth and available RF power, we seek a practical multi-tone waveform solution maximizing the isolation within these constraints.

***Reviewer Comment #4.** Page 7 bottom left...section about "to maximize isolation". (this is a suggestion and I defer how/if this should be addressed to the authors and the editor): The requirement of specific harmonics potentially precludes platforms without electro-optics from benefiting from the techniques outlined in this work. I think it would add a large amount of value to the paper to analyze the case where driving at an exact harmonic is not possible in the*

SI. A simple case to analyze could be using your optimized waveform in simulation and changing the 3rd harmonic to e.g. the "2.8-th" harmonic instead and seeing what the predicted isolation contrast becomes. This analysis would help generalize this technique to other platforms where acousto-optics is the preferred modulations strategy.

Author Response #4: We thank the reviewer for this insightful question. First, we would like to point out that when the exact integer harmonic is replaced by a non-integer frequency, a continuous temporal phase walk-off occurs between the fundamental and the higher-order tone. Because the waveform is no longer stationary, the strict destructive interference is periodically lost. As can be reasonably expected, modeling confirms that the worst degradation is at least on the same order as the isolation without the harmonic. More generally, considering the traveling wave nature of the modulation, a space-periodic phase detuning of the same type would arise over long travel distance if the traveling wave had a substantial dispersion, such that the fundamental and the harmonic acquire a space-variant phase shift. This is not a significant consideration in conventional co-planar RF waveguides here, which are only about one wavelength long for the fundamental. However, this may be of significant concern in some potential AO implementations where the length needed to impart sufficient modulation depth corresponds to many acoustic waves. To extend our method to such devices, assuming integer harmonics can be launched by a suitable coupler, presumably, the acoustic waves must be dispersion-engineered to avoid significant dispersion between the fundamental and harmonic(s). Unfortunately, a more detailed study of this case is well beyond the scope of the present work and would require additional careful analysis and validation. To explicitly clarify this issue for the readers, we have added the following to the manuscript.

> It is worth noting that this multi-tone modulation strategy relies on the exact integer harmonics. In platforms where generating exact integer harmonics is challenging, the continuous temporal phase walk-off between the non-integer tones can lead to a degradation in isolation.

***Reviewer Comment #5**. page 12 discussion on Insertion Loss: if the bonding process were to be perfected, what insertion loss do the authors believe would be achievable?*

Author Response #5: We thank the reviewer for raising this important question.

If the bonding process and fabrication imperfections were perfectly resolved, the insertion loss would be entirely dominated by the passive optical components. It would be limited only by the insertion loss of the directional couplers and the propagation loss of the waveguides such as sidewall scattering loss and material absorption loss. We would like to emphasize an important theoretical point regarding our design. In an ideal scenario where these passive scattering and absorption losses are minimized, the DRDI architecture itself possesses zero intrinsic insertion loss in the forward-transmitting direction. Unlike some conventional optical isolator designs that inherently suffer from a fundamental loss, the theoretical minimum insertion loss of our DRDI approach is 0 dB.

To make this fundamental advantage clear to the readers, we have expanded the following discussion on insertion loss of the revised manuscript.

> Assuming a perfect bonding process, the total insertion loss of the device would be dominated by the passive components. Importantly, in the ideal case where passive losses such as the insertion loss of the directional couplers and the waveguide propagation loss are minimized, the DRDI itself introduces zero intrinsic insertion loss in the forward direction.

Two questions involve equations and are provided in pdf and txt format.

[Comments from the additional PDF]

***Reviewer Comment #6:** The authors use phrases like "with up to N harmonics" and I think this is a bit confusing. If I understandcorrectly, N=3 harmonics actually means the first and third harmonic since even order harmonics are avoided.*

The authors should revisit how they describe the number of harmonics used. Perhaps something like "N is the number of odd harmonics".

Author Response #6: We fully agree with the reviewer about the terminology. The reviewer is absolutely correct that we strictly avoid even-order harmonics and only utilize odd-order tones. Therefore, *N* represents the highest harmonic order present in the waveform, not the total number of harmonic tones used. To eliminate any confusion, we have carefully revisited our terminology throughout the manuscript. We have replaced the phrase 'up to *N* harmonics' with 'odd harmonics up to the *N*-th order,' and explicitly defined *N* as the maximum odd-harmonic order. We thank the reviewer for pointing out. This significantly improves the precision of our manuscript.

Reviewer Comment #7:

Q: Can the authors include more information (in the SI) regarding the theory model of their device? Based on my guess for how it is done, some sort of transfer matrix or scattering matrix might be appropriate if the authors work in the time domain, e.g., transmission through the device given the setup in Fig. 1b is

$$\tau(t) = \begin{pmatrix} i/2 & 1/2 & i/2 & -1/2 \end{pmatrix} \begin{pmatrix} e^{i\phi_1(t)} & 0 & 0 & 0 \\ 0 & e^{i\phi_2(t)} & 0 & 0 \\ 0 & 0 & e^{i\phi_3(t)} & 0 \\ 0 & 0 & 0 & e^{i\phi_4(t)} \end{pmatrix} \begin{pmatrix} i/2 \\ 1/2 \\ i/2 \\ -1/2 \end{pmatrix}.$$

$$= \frac{1}{4}\left\{-e^{i\phi_1(t)} + e^{i\phi_2(t)} - e^{i\phi_3(t)} + e^{i\phi_4(t)}\right\}$$

I have neglected the role of the heaters for simplicity.

Author Response #7: We sincerely thank the reviewer for this excellent suggestion and for providing such a precise mathematical insight. We completely agree that explicitly showing this time-domain transfer matrix derivation greatly enhances the theoretical foundation and clarity of our paper. Following your constructive recommendation, we have added a new section to the Supplementary Information.

Reviewer Comment #8:

Q: In the main text, there is a sentence that says that "the integrated microheaters are not strictly necessary". However, I have attempted to trace the light in the PIC shown in Figure 1 with my postulated theory model, and I believe there is some subtlety introduced by the 1x4 and 4x1 couplers.

If we take my expression for transmitted light given originally in one of my previous questions,

$$\tau(t) = \frac{1}{4}\left\{-e^{i\phi_1(t)} + e^{i\phi_2(t)} - e^{i\phi_3(t)} + e^{i\phi_4(t)}\right\}$$

and apply $\phi_2(t) = -\phi_1(t)$ and $\phi_4(t) = -\phi_3(t)$, which holds true for all the times labeled in Fig. 2c, we get

$$\begin{aligned}\tau(t) &= \frac{1}{4}\left\{-e^{i\phi_1(t)} + e^{-i\phi_1(t)} - e^{i\phi_3(t)} + e^{-i\phi_3(t)}\right\} \\ &= \frac{1}{4}\left\{-\left(e^{i\phi_1(t)} - e^{-i\phi_1(t)}\right) - \left(e^{i\phi_3(t)} - e^{-i\phi_3(t)}\right)\right\} \\ &= -\frac{i}{2}\{\sin(\phi_1(t)) + \sin(\phi_3(t))\}\end{aligned}$$

Now we step through the different time instances in Figure 2e and 2c. At $t = T_0/32$, $\phi_3(T_0/32) = \phi_1(T_0/32) - \pi$,

$$\begin{aligned}\tau(T_0/32) &= -\frac{i}{2}\{\sin(\phi_1(T_0/32)) + \sin(\phi_1(T_0/32) - \pi)\} \\ &\equiv 0.\end{aligned}$$

This cancellation is what I would expect. At $t = 10T_0/32$, $\phi_3(10T_0/32) = -\phi_1(10T_0/32) - \pi$,

$$\begin{aligned}\tau(10T_0/32) &= -\frac{i}{2}\{\sin(\phi_1(10T_0/32)) + \sin(-\phi_1(10T_0/32) - \pi)\} \\ &= -i\sin(\phi_1(10T_0/32))\end{aligned}$$

In this region, the transmission is not identically zero, which goes against my expectation.

Figure 2 diagrammatically makes sense and I agree that it is probably the best way to illustrate the effect, but I believe there is some subtlety introduced by your 1x4 and 4x1 couplers that makes biasing with heaters a necessity to ensure the recombination phases are proper for DRDI. I believe the origin of this discrepancy in my model comes from directional couplers introducing phases in their cross terms. Or, it is possible my model is wrong. But in any case, I would like to see the theory model of your devices describing light propagation through the 1x4 couplers, through the modulators, and then through the 4x1 coupler, under modulation by a triangle wave put into the SI.

Since the effect you are demonstrating is dependent on the phases in each modulator in relation to one another, an accounting of the optical phases through the whole device is important for reproducibility.

Author Response #8: We appreciate the reviewer's thorough mathematical analysis of our device. The reviewer is correct that the cascaded directional couplers intrinsically introduce phase differences due to their cross-coupling terms, which would prevent perfect destructive interference if driven solely by the RF signals. When we originally stated that "integrated microheaters are not strictly necessary," our intention was to convey that the required static phase offsets (π phase shifts) could, in principle, be implemented passively during the fabrication process by designing the waveguides with asymmetric lengths (e.g. using omega-shaped optical delay lines). However, we fully agree with the reviewer that our original phrasing was ambiguous and confusing in the context of an active device. To eliminate this confusion, we have revised the misleading sentence in the main text. Furthermore, following your suggestion, we have also expanded our transfer matrix model in the Supplementary Information. We explicitly introduced the static phase offset terms (θ) provided by the heaters (or passive delay lines) and mathematically demonstrated how they

perfectly compensate for the intrinsic coupler phases to achieve the ideal in-phase condition and finally complete destructive interference at any time. The following is the new section in the Supplementary Information.

As illustrated in the schematics Figure 1, the 1×4 splitting section consists of cascaded directional couplers. Let the state of the complex optical electric field in the four waveguide arms (ordered from top to bottom: 3, 2, 1, 4) be represented by a column vector. The input light enters through the port 1, giving the initial state vector $E_{in} = (0, 0, 1, 0)^T$.

The splitting process occurs in two stages. The first stage (M_1) couples the inner waveguides (ports 1 and 2), and the second stage (M_2) consists of two parallel couplers mixing the outer and inner waveguides. The transfer matrices for these stages are

$$M_1 = \begin{pmatrix} 1 & 0 & 0 & 0 \\ 0 & \frac{1}{\sqrt{2}} & \frac{i}{\sqrt{2}} & 0 \\ 0 & \frac{i}{\sqrt{2}} & \frac{1}{\sqrt{2}} & 0 \\ 0 & 0 & 0 & 1 \end{pmatrix}, \quad M_2 = \frac{1}{\sqrt{2}} \begin{pmatrix} 1 & i & 0 & 0 \\ i & 1 & 0 & 0 \\ 0 & 0 & 1 & i \\ 0 & 0 & i & 1 \end{pmatrix} \tag{S4}$$

The optical electric field distribution entering the phase modulators is obtained by multiplying these matrices with the input vector

$$V_{split} = M_2 M_1 E_{in} = \begin{pmatrix} -1/2 \\ i/2 \\ 1/2 \\ i/2 \end{pmatrix} \tag{S5}$$

The four electro-optic phase modulators introduce time-varying phase shifts with phase offsets by the heaters (or passive optical delay lines), which can be represented by a diagonal matrix $M_\phi(t)$ with the optical channel numbers as specified in Figure 2b. Finally, the output combiner section is structurally symmetric to the splitter. Therefore, the total complex transmission at the port 1, $\tau(t)$, is given by projecting the combined field

$$\tau(t) = \begin{pmatrix} -1/2 & i/2 & 1/2 & i/2 \end{pmatrix} \begin{pmatrix} e^{i(\phi_1(t)+\theta_1)} & 0 & 0 & 0 \\ 0 & e^{i(\phi_2(t)+\theta_2)} & 0 & 0 \\ 0 & 0 & e^{i(\phi_3(t)+\theta_3)} & 0 \\ 0 & 0 & 0 & e^{i(\phi_4(t)+\theta_4)} \end{pmatrix} \begin{pmatrix} -1/2 \\ i/2 \\ 1/2 \\ i/2 \end{pmatrix} \tag{S6}$$

$$\tau(t) = \frac{1}{4} \left\{ e^{i(\phi_1(t)+\theta_1)} - e^{i(\phi_2(t)+\theta_2)} + e^{i(\phi_3(t)+\theta_3)} - e^{i(\phi_4(t)+\theta_4)} \right\} \tag{S7}$$

The negative signs in Equation (S7) represent the intrinsic π phase shifts introduced by the cross-coupling terms of the cascaded directional couplers. To ensure perfect destructive interference driven strictly by the RF waveforms, we bias the device to an in-phase condition by applying appropriate static phase offsets via the microheaters. Alternatively, this static phase compensation can be achieved passively by designing the waveguides with asymmetric lengths. Specifically, by setting $\theta_1 = 0$, $\theta_2 = \pi$, $\theta_3 = 0$, and $\theta_4 = \pi$, we can achieve in-phase condition between the four optical channels.

$$\tau(t) = \frac{1}{4} \left\{ e^{i\phi_1(t)} + e^{i\phi_2(t)} + e^{i\phi_3(t)} + e^{i\phi_4(t)} \right\} \tag{S8}$$

Furthermore, our device operates in a push-pull configuration, where the respective channels of the dual-drive Mach-Zehnder modulators provide equal and opposite phase shifts. This dictates that $\phi_2(t) = -\phi_1(t)$ and $\phi_4(t) = -\phi_3(t)$ for all times t. Substituting these conditions into Equation (S8) yields

$$\tau(t) = \frac{1}{4} \left\{ e^{i\phi_1(t)} + e^{-i\phi_1(t)} + e^{i\phi_3(t)} + e^{-i\phi_3(t)} \right\} \tag{S9}$$

Using Euler's formula, this equation simplifies to real-valued cosine functions

$$\tau(t) = \frac{1}{2} \left\{ \cos(\phi_1(t)) + \cos(\phi_3(t)) \right\} \tag{S10}$$

Based on Equation (S10), we can explicitly verify the perfect destructive interference at the specific time instances highlighted in Figure 2e.

First, in the case where Channel 1 and Channel 3 cancel each other out at $t = T_0/32$ and $t = 19T_0/32$, the phase relationship is $\phi_3(t) = \phi_1(t) - \pi$. Substituting this into Equation (S10) yields obvious signal cancellation

$$\tau(t) = \frac{1}{2}\{\cos(\phi_1(t)) + \cos(\phi_1(t) - \pi)\} = \frac{1}{2}\{\cos(\phi_1(t)) - \cos(\phi_1(t))\} = 0 \tag{S11}$$

Second, in the case where Channel 1 and Channel 4 cancel each other out at $t = 10T_0/32$ and $t = 26T_0/32$, the required phase relationship is $\phi_4(t) = \phi_1(t) - \pi$. Given the intrinsic push-pull condition of the device ($\phi_4(t) = -\phi_3(t)$), this relation is mathematically equivalent to $\phi_3(t) = -\phi_1(t) + \pi$. Substituting this condition into Equation (S10) also yields perfect cancellation

$$\tau(t) = \frac{1}{2}\{\cos(\phi_1(t)) + \cos(-\phi_1(t) + \pi)\} = \frac{1}{2}\{\cos(\phi_1(t)) - \cos(\phi_1(t))\} = 0 \tag{S12}$$

These derivations mathematically confirm that our phase-compensated theoretical model completely guarantees $\tau(t) = 0$ in both cancellation scenarios, supporting the complete theoretical isolation of our work.

Reviewer #3:

This manuscript reports a magnet-free integrated optical isolator based on a four-channel electro-optic traveling-wave architecture implementing dynamic rotating destructive interference (DRDI). The device is realized on a SiN/TFLN platform and demonstrates about 30 dB isolation near 789.7 nm, more than 24 dB isolation across a 30 nm span with heater-assisted tuning, and simultaneous more than 20 dB isolation for two lasers across a 10 nm range. Overall, I find the work novel, significant, and potentially suitable for publication in Nature Photonics.

The paper addresses an important long-standing challenge in integrated photonics: achieving broadband non-magnetic on-chip isolation without magneto-optic materials. The DRDI concept is elegant and well explained, and the experimental results are compelling. I also view the dual-laser experiment as an important strength, because it moves the paper beyond a narrow single-wavelength proof of concept and supports the broader relevance of the approach.

Author Response: We sincerely thank the reviewer for their highly positive evaluation of our manuscript and for recognizing the novelty. We also deeply appreciate the constructive feedback provided to further strengthen the manuscript. We have carefully addressed each comment and revised the manuscript accordingly. Detailed responses to your suggestions are provided below.

Reviewer Comment #1: *I have only a few suggestions to strengthen the manuscript further. First, the broadband claim should be phrased more carefully, since the best 30 nm result relies on active heater tuning at different wavelengths; this should be distinguished more clearly from fixed-bias operation. Second, the forward-transmission advantage should be quantified more clearly in the main text, since this is one of the key conceptual benefits of the approach. The manuscript states that the forward spectrum under RF is almost identical to the RF-off case, indicating no added loss or frequency shift, and this point deserves a more explicit quantitative presentation.*

Author Response #1: We completely agree that the broadband claim and the forward-transmission advantage should be presented more explicitly. In the revised manuscript's abstract, introduction and discussion sections, we have updated the text to explicitly state the 30 nm range isolation achieved via wavelength-dependent thermo-optic tuning, and the 10 nm range isolation by fixed-bias experiment. Furthermore, we have added the following quantitative analysis to the main text to highlight the low intrinsic-penalty advantage of our approach.

> Importantly, the DRDI scheme preserves the transmission of the forward-propagating light without introducing additional measurable loss (Extended Data Fig. 1). To rigorously quantify this, we performed a statistical comparison of the forward transmission spectra in the presence and absence of the RF drive. Across a 30 nm wavelength span, the application of the RF drive introduces a negligible excess insertion loss of 0.023 dB ± 0.018 dB under a fixed heater bias, and 0.009 dB ± 0.056 dB when the heaters are actively adjusted for each wavelength. These variations are statistically indistinguishable from zero within the tight bounds of small experimental measurement uncertainties.

Reviewer Comment #2: *I also suggest updating the comparison table to include the recent Nature Photonics demonstration of an integrated terahertz-bandwidth non-magnetic isolator (https://www.nature.com/articles/s41566-025-01663-8). That work is directly relevant to the present manuscript and would provide a useful recent benchmark for bandwidth, insertion loss, and mechanism-level comparison.*

Author Response #2: We sincerely thank the reviewer for bringing this outstanding and highly relevant recent work to our attention. To include this recent work, we have updated our comparison table (Extended Data Table 1) and the main text.

In summary, this is a strong and original manuscript that makes an important contribution to integrated nonreciprocal photonics. I am supportive of publication in Nature Photonics after minor revision.

Reviewer #1:
Remarks to the Author:
The authors have answered all questions and comments from the Reviewers. The quality of the paper after the revision has been much improved. I would recommend publication of the manuscript in Nature Photonics, and I have no further questions for the authors.

We sincerely thank the Reviewer for their time and effort reviewing our work.

Reviewer #2:
Remarks to the Author:
The Authors' response and updates to the manuscript have addressed all our suggestions. We recommend the manuscript for publication.

Sunil Bhave and Scott Kenning (Graduate Student)

We sincerely thank Prof. Bhave and Mr. Kenning for their time and effort reviewing our work.

Reviewer #3:
Remarks to the Author:
I have checked the revised manuscript and response, I believe it’s good for publication.

We sincerely thank the Reviewer for their time and effort reviewing our work.
